# Parallel roles of transcription factors dFOXO and FER2 in the development and maintenance of dopaminergic neurons

**DOI:** 10.1371/journal.pgen.1007271

**Published:** 2018-03-12

**Authors:** Damla Tas, Luca Stickley, Federico Miozzo, Rafael Koch, Nicolas Loncle, Virginie Sabado, Bettina Gnägi, Emi Nagoshi

**Affiliations:** 1 Department of Genetics and Evolution, Sciences III, University of Geneva, 30 Quai Ernest-Ansermet, Geneva-4, CH, Switzerland; 2 Institute of Cell Biology, University of Bern, Baltzerstrasse 4, Bern, CH, Switzerland; 3 Institute of Genetics and Genomics in Geneva (iGE3), University of Geneva, Geneva, Switzerland; Stanford University School of Medicine, UNITED STATES

## Abstract

Forkhead box (FOXO) proteins are evolutionarily conserved, stress-responsive transcription factors (TFs) that can promote or counteract cell death. Mutations in FOXO genes are implicated in numerous pathologies, including age-dependent neurodegenerative disorders, such as Parkinson’s disease (PD). However, the complex regulation and downstream mechanisms of FOXOs present a challenge in understanding their roles in the pathogenesis of PD. Here, we investigate the involvement of FOXO in the death of dopaminergic (DA) neurons, the key pathological feature of PD, in *Drosophila*. We show that dFOXO null mutants exhibit a selective loss of DA neurons in the subgroup crucial for locomotion, the protocerebral anterior medial (PAM) cluster, during development as well as in adulthood. PAM neuron-targeted adult-restricted knockdown demonstrates that dFOXO in adult PAM neurons tissue-autonomously promotes neuronal survival during aging. We further show that dFOXO and the bHLH-TF 48-related-2 (FER2) act in parallel to protect PAM neurons from different forms of cellular stress. Remarkably, however, dFOXO and FER2 share common downstream processes leading to the regulation of autophagy and mitochondrial morphology. Thus, overexpression of one can rescue the loss of function of the other. These results indicate a role of dFOXO in neuroprotection and highlight the notion that multiple genetic and environmental factors interact to increase the risk of DA neuron degeneration and the development of PD.

## Author summary

PD, mainly characterized by a progressive loss of dopaminergic neurons in the substantia nigra (SN), is the most prevalent neurodegenerative movement disorder affecting more than 6 million people worldwide. Despite the discovery of several genes linked to familial PD, our understanding of its pathogenesis remains limited, as approximately 90% of the PD cases are sporadic with no apparent genetic linkage. Genome-wide expression studies have implicated the stress-responsive TF FOXO in PD. However, the exact role of FOXO in the survival of DA neurons and PD pathogenesis is still poorly understood. Here, we use fruit flies to address the role of FOXO in the maintenance of DA neurons. dFOXO (*Drosophila* FOXO) null mutants show a progressive loss of DA neurons in the subgroup essential for locomotion, a phenotype identical to that of *Fer2* mutants. Remarkably, dFOXO and FER2 act in parallel pathways to protect PAM neurons from different cellular stressors, but both pathways contribute to the regulation of autophagy and mitochondrial biology. These results demonstrate that dFOXO is required for the maintenance of DA neurons important for locomotion and shed new light on the molecular mechanisms underpinning the complex gene-environment interactions affecting DA neuron survival and PD pathogenesis.

## Introduction

Parkinson’s disease (PD) is the most prevalent neurodegenerative movement disorder characterized by the progressive loss of dopaminergic (DA) neurons in the substantia nigra (SN). Interactions between genetic and environmental factors are crucial to PD pathogenesis [1, 2]. The discovery of monogenic gene mutations associated with rare familial PD and genome-wide association studies (GWASs) for sporadic PD have advanced the identification of genetic risk factors [3–5]. In addition, a number of environmental factors including oxidative stress, pesticide and herbicide exposure, heavy metal exposure, and even nutrients [6, 7] have been shown to contribute to the etiology of PD. However, the mechanisms underlying the gene-environment interactions that enhance the risk of PD development are still not fully understood.

*Drosophila* offers the opportunity to investigate genetic and molecular mechanisms of PD pathogenesis *in vivo* in a relatively short period of time and at single-cell resolution, due to the advanced genetic tools and the relatively simple nervous system [8]. Fly brain DA neurons form approximately a dozen clusters projecting to different brain areas [9]. DA neurons in the protocerebral anterior medial (PAM) cluster are required for the startle-induced climbing behavior, which is defective in various PD models [10]. Several genetic fly models of PD have been generated by introducing mutations or by overexpressing mutant human genes linked to familial PD or their homologs (reviewed in [11]). Although controversies remain regarding the extent to which these PD models display a genuine loss of DA neurons [12], they provide insights into the mechanisms of PD pathogenesis. In particular, mitochondrial dysfunction and elevated oxidative stress–two major cellular pathogenic characteristics in PD patients’ brains [13]–have been observed in many genetic fly models of PD [14–18]. Despite these advances in the study of familial PD pathogenesis, the mechanistic understanding of sporadic PD, which accounts for approximately 90% of PD cases, is lagging behind.

We have previously shown that the *p48-related-2* (*Fer2*) gene encoding a bHLH-transcription factor (TF) is expressed in the PAM cluster DA neurons and required for their development and survival [19]. The *Fer2*^*2*^ hypomorph mutant displays a progressive loss of PAM neurons and a concomitant impairment in the startle-induced climbing ability. Moreover, in *Fer2*^*2*^ flies, the levels of reactive oxygen species (ROS) are elevated in the brains and mitochondrial morphology is impaired specifically in PAM neurons. Acute oxidative stress upregulates *Fer2* mRNA levels and induces rapid loss of PAM neurons in *Fer2*^*2*^ mutants. These results indicate that *Fer2* protects PAM neurons from oxidative stress [19]. In addition to the marked phenotypic similarities between *Fer2*^*2*^ mutant flies and PD patients, polymorphisms in *PTF1a* (*p48*), a mammalian homolog of *Fer2*, are also associated with sporadic PD [3, 20]. Taken together, these lines of evidence suggest that the *Fer2*^*2*^ mutant provides a unique tool to study the mechanistic links between oxidative stress, mitochondrial dysfunction and genetic risk factors critical for sporadic PD.

Emerging evidence implicates the decline in protein homeostasis (proteostasis) in various age-related neurodegenerative disorders, including PD [21]. The FOXO family plays pivotal roles in proteostasis by regulating autophagy and the ubiquitin-proteasome system [22, 23]. A gene expression analysis of brain tissues from PD patients implicated *FOXO1* in PD pathogenesis [24]. In addition, studies in mice showed that FOXA1 and A2 are required for the maintenance of adult midbrain DA neurons [25–27], and FOXO3 activity modulates the α-synuclein toxicity and survival of DA neurons in the SN [28]. The sole homolog of FOXOs in flies, dFOXO, has been reported to both negatively and positively affect DA neuron survival in familial PD models. Whereas dFOXO overexpression has been shown to protect DA neurons in *Pink1* null mutants [29], other studies have shown that induction of apoptosis by dFOXO mediates DA neuron demise in *DJ-1β* mutants and in dLRRK-misexpressed flies [30, 31]. These results suggest a role for FOXO factors in sporadic or familial PD pathogenesis and warrant further analysis to tease apart how FOXOs regulate the survival of DA neurons.

In this study, we reevaluate the role of dFOXO in DA neuron maintenance. We demonstrate that *dfoxo* null mutants are phenotypically similar to *Fer2*^*2*^ mutants in that both show mild loss of DA neurons in the PAM cluster during development, followed by their progressive degeneration in adulthood and locomotor deficits. Genetic interaction assays reveal that *dfoxo* and *Fer2* act in parallel pathways to control development and survival of PAM neurons. In adults, *Fer2* is required for the survival of PAM neurons under acute oxidative stress and for preventing overproduction of ROS in the brain but *dfoxo* is not, indicating that *dfoxo* and *Fer2* pathways are activated by different stress conditions. Intriguingly, however, *dfoxo* and *Fer2* compensate for each other’s loss of function. This is in part because they share common downstream mechanisms leading to the regulation of autophagy and mitochondrial morphology in PAM neurons. These results demonstrate the complexity in the gene-environment interactions affecting DA neuron survival and highlight the multifactorial nature of PD etiology.

## Results

### dFOXO is required for the development and survival of PAM neurons

**Fig 1:**
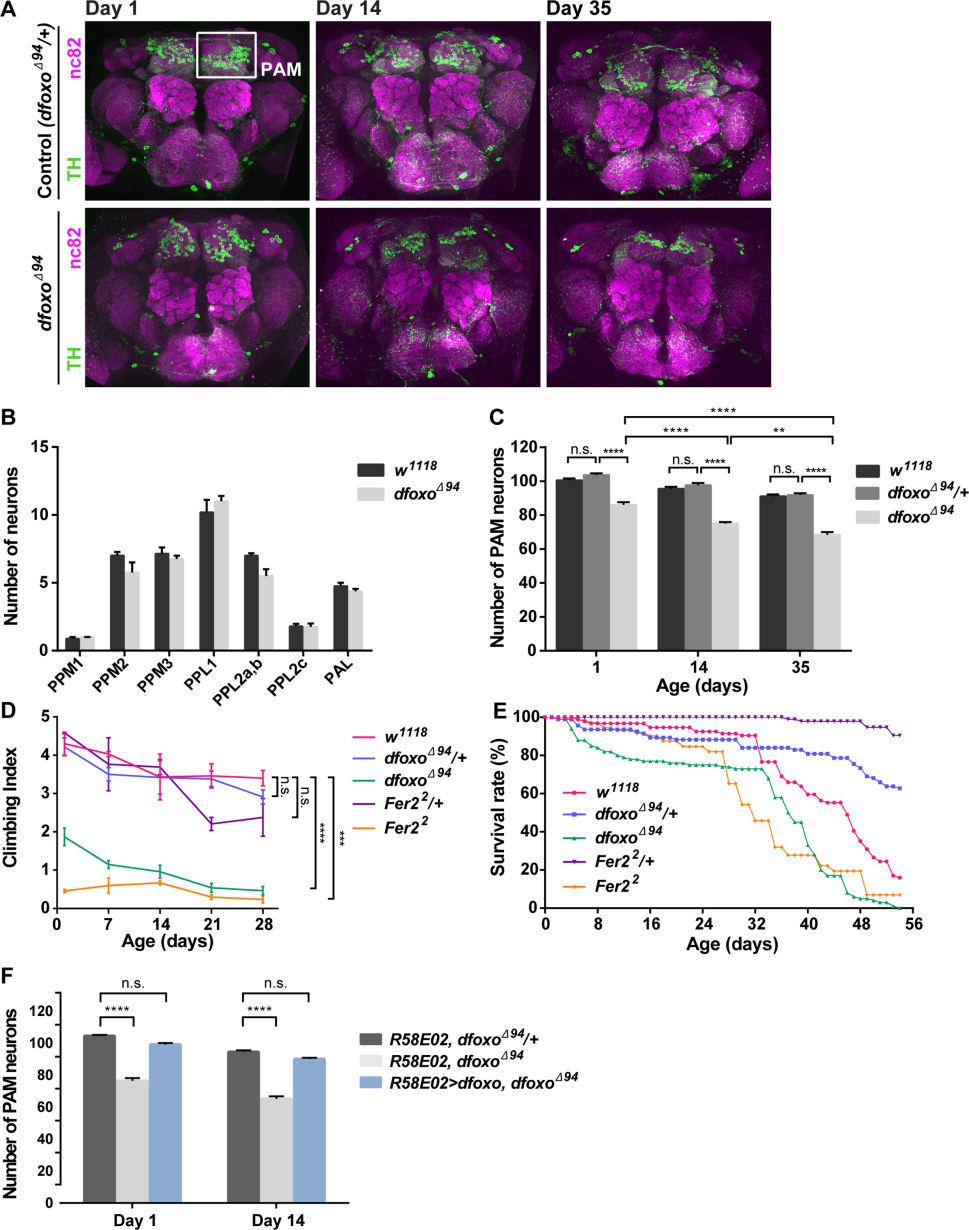
dFOXO is cell-autonomously required for the development and maintenance of DA neurons in the PAM cluster. (A) DA neurons in the brains detected by anti-TH staining (green) in *dfoxo*^*Δ94*^*/+* and *dfoxo*^*Δ94*^ flies of the indicated ages. Neuropil was counterstained with the anti-nc82 antibody (magenta). (B) The numbers of DA neurons at day 14 in each cluster except for the PAM cluster. No significant differences are found between *w*^*1118*^ and *dfoxo*^*Δ94*^ in any of these clusters (n = 6–8). (C) Quantification of PAM neurons in *w*^*1118*^, *dfoxo*^*Δ94*^ heterozygous and homozygous flies. The number of PAM neurons in *dfoxo*^*Δ94*^ is significantly reduced compared with *dfoxo*^*Δ94*^*/+* and *w*^*1118*^ at all ages (n = 18–24). (D) Startle-induced climbing response assayed weekly. *dfoxo*^*Δ94*^ and *Fer2*^*2*^ flies exhibit a significantly impaired climbing ability. (***p< 0.001 and ****p<0.0001, Mann–Whitney U-test). (E) Lifespan of *dfoxo*^*Δ94*^ and *Fer2*^*2*^ homozygous flies is significantly reduced compared to that of their heterozygous control (p<0.0001, log-rank test) and *w*^*1118*^ (p<0.0001, log-rank test). (F) Rescue of the PAM neuron counts in *dfoxo*^*Δ94*^ mutants by driving expression of *UAS-dfoxo* with *R58E02-GAL4* (n = 15–22). Mean counts of neurons per hemisphere, error bars represent SEM. n.s., not significant, **p<0.01 and ****p<0.0001 by ANOVA.

To determine whether dFOXO is involved in the control of brain DA neuron viability, we assessed the integrity of DA neurons by immunostaining with anti-tyrosine hydroxylase (TH) antibodies in *dfoxo* null mutants, *dfoxo*^*Δ94*^ [32]. We observed approximately 15% fewer DA neurons in the PAM cluster in *dfoxo*^*Δ94*^ mutants compared to *w*^*1118*^ and *dfoxo*^*Δ94*^/+ flies at 1 day old. The number of PAM neurons in *dfoxo*^*Δ94*^ mutants continued to decrease with age, resulting in an approximate 20% loss between 1- and 35-day-old flies. The number of PAM neurons in *dfoxo*^*Δ94*^*/+* was not different from that of *w*^*1118*^ at least up to 35 days of age (Fig 1A and 1C). Interestingly, no other DA neuron clusters were affected in *dfoxo*^*Δ94*^ mutants (Fig 1B). Furthermore, to distinguish the loss of TH expression and neuronal loss, we drove the expression of nuclear-targeted RFP (RedStinger) with *R58E02-GAL4*, which is expressed in pupal and adult PAM neurons [33, 34], in *dfoxo*^*Δ94*^*/+* and *dfoxo*^*Δ94*^ flies. There were fewer RedStinger-positive PAM neurons in *dfoxo*^*Δ94*^ flies than in *dfoxo*^*Δ94*^*/+* both at 1 day and 14 days of age, with a greater difference at 14 days (S1A and S1B Fig). Therefore, fewer neurons are formed and survive in adulthood in the PAM cluster in *dfoxo*^*Δ94*^ mutants compared to *dfoxo*^*Δ94*^*/+*.

A subset of PAM neurons is necessary for the control of locomotion [10]. Consistently, *Fer2* mutants, which display a selective loss of PAM neurons, show the locomotor deficits characteristic of *Drosophila* PD models [19]. Similarly, *dfoxo*^*Δ94*^ but not *dfoxo*^*Δ94*^*/+* flies showed significant locomotor impairments in a startle-induced climbing assay (Fig 1D). We also observed that the lifespan of *dfoxo*^*Δ94*^ mutants and *Fer2*^*2*^ flies was reduced compared to that of their heterozygous controls and wild-type flies, which also resembles the phenotypes of other *Drosophila* PD models [14, 18, 35–37] (Fig 1E).

The *dfoxo* gene is expressed in many tissues at varying levels, with most prominent expression in the larval and adult fat body (http://www.flyatlas.org/). RNA-seq analysis of isolated PAM neurons confirmed the expression of *dfoxo* in PAM neurons (S2 Fig). We next sought to conduct genetic rescue of *dfoxo*^*Δ94*^ mutants using the GAL4/UAS system. To this end, we analyzed the expression of PAM neuron-driver *R58E02-GAL4* and *Fer2-GAL4* in the fat body by driving *UAS-GFP-NLS* (S3 Fig). Unexpectedly, we found that *R58E02-GAL4* was expressed in a small fraction of abdominal fat body stained with Nile Red in 1-day-old but not in 21-day-old flies (S3A Fig). Although no *R58E02-GAL4* expression was apparent in the fat body of 3rd instar larvae (S3B Fig), because the larval fat body survives through metamorphosis into the first few days of adulthood [38], these results suggest that *R58E02-GAL4* is expressed in some fat body cells between the larval and pupal stages at least transiently. In contrast, virtually no *Fer2-GAL4* expression was detected in the abdominal fat body of both 1- and 21-day-old flies or in the larval fat body (S3A and S3B Fig). No expression of either driver was apparent in the head fat body. Therefore, taking the *dfoxo* expression in the fat body into account, we used *R58E02-GAL4* to re-express *dfoxo* in *dfoxo*^*Δ94*^ mutants and examined the PAM neuron counts in adult flies. As shown in Fig 1F, expression of *dfoxo* with *R58E02-GAL4* rescued the development and degeneration of PAM neurons in *dfoxo*^*Δ94*^ flies.

Taken together, these results indicate that dFOXO is required for the proper development and maintenance of DA neurons during aging selectively in the PAM cluster. In agreement with these findings, *dfoxo* null mutants show PD-like locomotor impairments.

### dFOXO in adult PAM neurons promotes neuronal survival during aging tissue-autonomously

**Fig 2:**
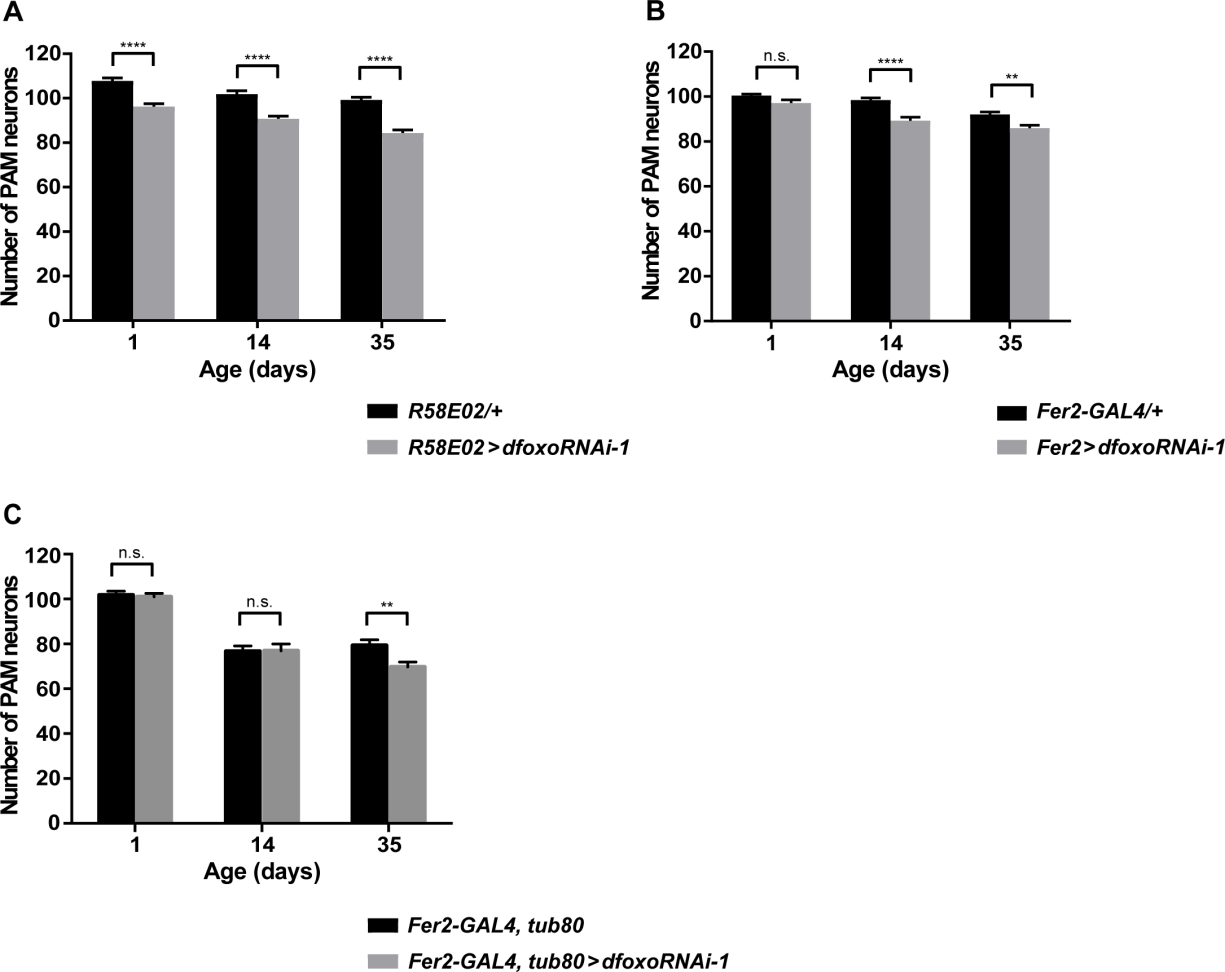
dFOXO is tissue-autonomously required for the survival of PAM neurons. (A-C) Quantification of PAM neurons detected by anti-TH staining following *dfoxo* knockdown. Mean counts of neurons per hemisphere, error bars represent SEM. **p<0.01, ****p<0.0001 by ANOVA. (A) *dfoxo* constitutive knockdown with *R58E02-GAL4* (n = 18–20). (B) *dfoxo* constitutive knockdown with *Fer2-GAL4* (n = 18–22) (C) *dfoxo* adult-restricted knockdown with *Fer2-GAL4*, *tub-GAL80*^*ts*^ (n = 24–36).

We next asked whether the loss of PAM neurons in *dfoxo*^*Δ94*^ is a tissue-autonomous effect of *dfoxo* deficiency. We addressed this question by tissue-specific *dfoxo* knockdown using *R58E02-GAL4* and *Fer2-GAL4*, because both drivers are commonly expressed in PAM neurons. When *UAS-dfoxo RNAi-1* (v30557) [39] was driven with *R58E02-GAL4*, the PAM neuron count was reduced already at 1 day old and continued to decrease thereafter (Fig 2A). Knockdown of *dfoxo* with 2 other RNAi lines, *UAS-dfoxo RNAi-2* (BDSC #32427) and *-3 (*BDSC *#32993*) [40], driven by *R58E02-GAL4* similarly resulted in the loss of PAM neurons in 1-day-old and additional loss in 14-day-old flies (S4 Fig). In contrast, when *UAS-dfoxo RNAi*-*1* was driven with *Fer2-GAL4*, significant loss of PAM neurons was observed only in the flies of 14 days old or older (Fig 2B). These results suggest that silencing *dfoxo* within PAM neurons leads to their progressive loss in adulthood, whereas reduced *dfoxo* expression in other tissues that express *R58E02-GAL4* but not *Fer2-GAL4*, probably including the larval fat body (S3A Fig), impairs the development of PAM neurons.

Since both *Fer2-GAL4* and *R58E02-GAL4* are also expressed in the pupal PAM neurons [19, 33], we next wanted to examine whether sustained expression of *dfoxo* in adulthood is tissue-autonomously required for the maintenance of PAM neurons during aging. To this end, we knocked down *dfoxo* in PAM neurons only during adulthood by driving *UAS-dfoxo RNAi-1* with the combination of *Fer2-GAL4* and a temperature-sensitive GAL80 expressed under the control of the *tubulin* promoter (*tubulin-GAL80*^*ts*^) [41]. The flies were reared at 18°C until eclosion and the newly eclosed flies were cultured at 29°C to induce the expression of *dfoxo RNAi*. This adult-restricted *dfoxo* knockdown caused a significant loss of PAM neurons in 35-day-old but not in younger flies (Fig 2C). These results indicate that *dfoxo* expressed in adulthood has a tissue-autonomous role in maintaining PAM neurons during aging. Nevertheless, the late onset of PAM neurodegeneration by adult-specific knockdown compared to constitutive knockdown suggest that loss of *dfoxo* expression during development has lasting negative impact on the viability of PAM neurons throughout adulthood.

### dFOXO and FER2 act in parallel pathways to promote survival of PAM neurons

**Fig 3:**
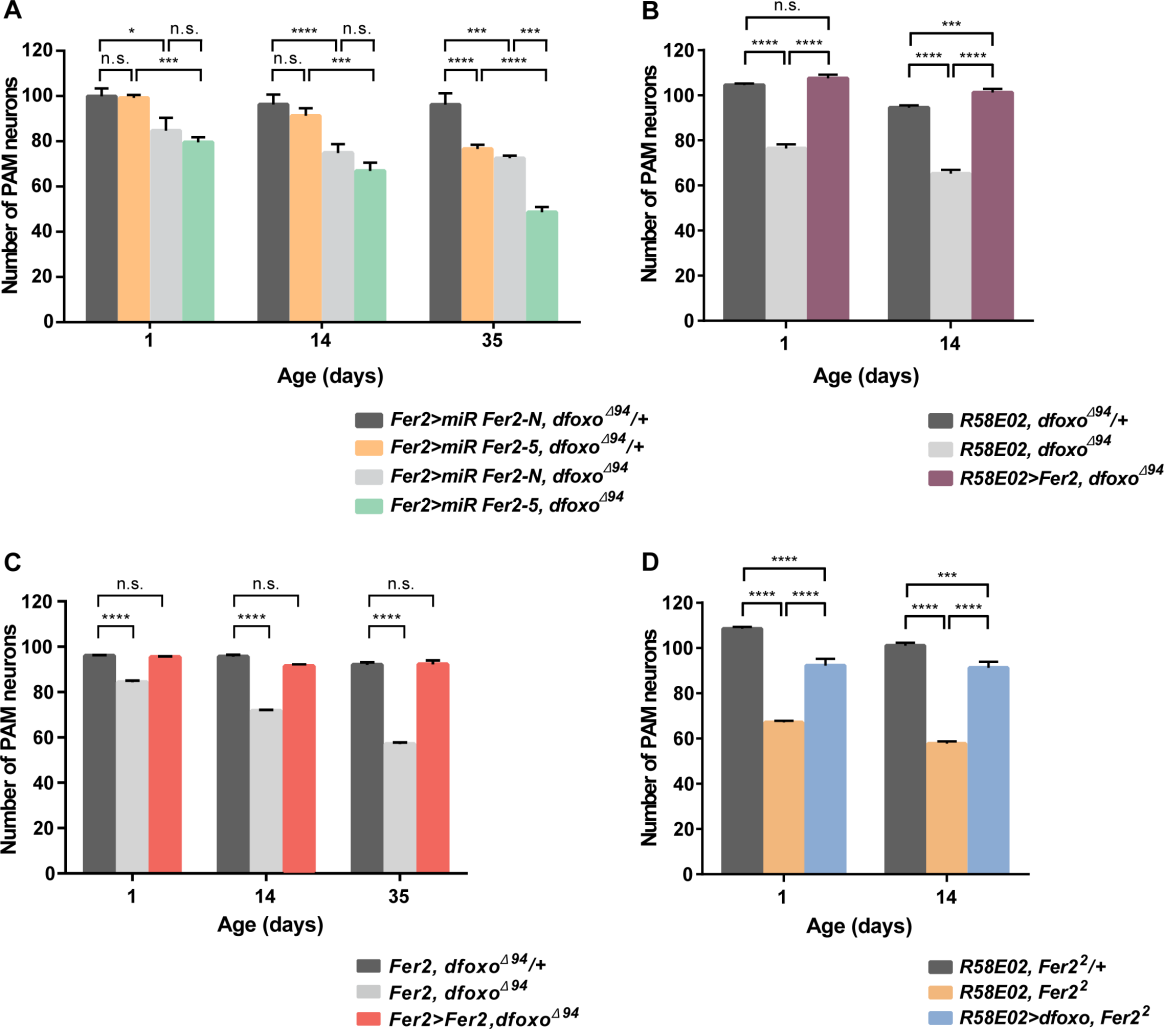
*dfoxo* and *Fer2* act in parallel compensatory pathways to maintain PAM neurons. (A-D) Quantification of PAM neurons detected by anti-TH staining in the indicated genotypes. Mean counts of neurons per hemisphere, error bars represent SEM. *p<0.05, *** p<0.001, **** p<0.0001 by ANOVA. (A) *Fer2* knockdown and *dfoxo* ^*Δ94*^ additively reduce the number of PAM neurons (n = 8–20). (B) Rescue of PAM neuron loss in *dfoxo*^*Δ94*^ by the expression of *Fer2* with *R58E02-GAL4* (n = 19–22). (C) Rescue of PAM neuron loss in *dfoxo*^*Δ94*^ by expressing *Fer2* using *Fer2-GAL4* (n = 12–26). (D) Rescue of PAM neuron loss in *Fer2*^*2*^ mutants by the expression of *dfoxo* with *R58E02-GAL4* (n = 12–22).

The findings that *dfoxo*^*Δ94*^ and *Fer2*^*2*^ mutants are phenotypically similar suggest a possible genetic interaction between *dfoxo* and *Fer2*. We therefore combined loss-of-function of both *dfoxo* and *Fer2* to determine whether dFOXO and FER2 act in linear or parallel pathways for controlling PAM neuron viability. We have previously shown that knockdown of *Fer2* with micro RNA against *Fer2* (*miR Fer2-5*) expressed with *Fer2-GAL4* causes adult-onset progressive loss of PAM neurons in wild-type flies, whereas negative control micro RNA (*miR Fer2-N*) has no effect [19]. Consistent with previous results, *miR Fer2-5* expression in the *dfoxo*^*Δ94*^*/+* background led to the adult-onset, age-dependent loss of PAM neurons, resulting in the loss of over 20 neurons in 35 days. *miR Fer2-N* expression had no effect on the PAM neuron number in the *dfoxo*^*Δ94*^*/+* background. *Fer2* knockdown with *miR Fer2-5* in *dfoxo*^*Δ94*^ homozygotes significantly reduced the number of PAM neurons, resulting in approximately 20 neurons fewer at day 35 compared with that in *dfoxo*^*Δ94*^ alone. Therefore, *Fer2* knockdown additively but not synergistically reduced the number of PAM neurons in *dfoxo*^*Δ94*^ flies (Fig 3A). These results preclude the possibility that *dfoxo* and *Fer2* act in a linear pathway. As in *dfoxo*^*Δ94*^ mutants, the integrity of PAL neurons was not affected by *dfoxo-Fer2* double loss-of-function (S5 Fig), suggesting that genetic interaction of *dfoxo* and *Fer2* selectively affects the viability of PAM neurons.

To better understand the functional relationship between *dfoxo* and *Fer2*, we next tested whether *Fer2* overexpression can rescue the phenotypes of *dfoxo* loss-of-function or vice versa. The expression of *UAS-Fer2-FLAG* with *R58E02-GAL4* or *Fer2-GAL4* completely rescued the development and subsequent loss of PAM neurons in *dfoxo*^*Δ94*^ (Fig 3B and 3C). Conversely, overexpression of *dfoxo* with *R58E02-*GAL4 partially rescued the development of PAM neurons in *Fer2*^*2*^ mutants and prevented their further loss in adulthood (Fig 3D).

Importantly, *Fer2* mRNA levels were unaffected in *dfoxo*^*Δ94*^ mutants (S6C Fig). Furthermore, by performing chromatin immunoprecipitation followed by sequencing (ChIP-seq) for FER2, we found that *dfoxo* was not a direct transcriptional target of FER2 (S6D Fig). Therefore, the common protective effect of *dfoxo* and *Fer2* on PAM neurons was mediated indirectly via their downstream genetic programs but not by the mutual transcriptional regulation. Taken together, these results suggest that *dfoxo* and *Fer2* act in parallel compensatory pathways in the development and maintenance of PAM neurons.

Consistent with the role of PAM neurons in controlling locomotion, the heterologous PAM neuron rescue using *R58E02-GAL4* significantly improved the climbing ability of *dfoxo*^*Δ94*^ and *Fer2*^*2*^ flies (S6A Fig). However, whereas *R58E02-GAL4* combined with *UAS-Fer2* extended the lifespan of *dfoxo*^*Δ94*^ flies, *UAS-dfoxo* did not increase the lifespan of *dfoxo*^*Δ94*^ and even reduced the lifespan of *Fer2*^*2*^ flies (S6B Fig). Therefore, the role of *dfoxo* and *Fer2* in longevity regulation is not linked to the viability of PAM neurons. The negative effect of *dfoxo* expressed with *R58E02-GAL4* on lifespan may be related to the expression patterns of this driver, which is not identical to that of endogenous *dfoxo* or *Fer2*.

### dFOXO and FER2 protect PAM neurons under different stress conditions

To corroborate the finding that dFOXO and FER2 act in parallel pathways, we next sought to delineate the upstream regulation of dFOXO and FER2. Transcriptional activity of FOXO factors is controlled by various environmental and metabolic stressors (reviewed in [22]). Oxidative stress activates FOXO TFs through Jun N-terminal kinase (JNK) signaling [42, 43]. On one hand, JNK/dFOXO signaling contributes to the suppression of oxidative stress-induced mitochondrial dysfunction and lethality in *Drosophila* [44]. On the other hand, dFOXO mediates apoptosis of DA neurons under oxidative stress triggered by *DJ-1β* mutation [30]. Thus, dFOXO may either promote or prevent cell death depending on the oxidative stress levels and genetic background. We therefore examined whether PAM neurons are more sensitive to oxidative stress in *dfoxo*^*Δ94*^ mutants.

**Fig 4:**
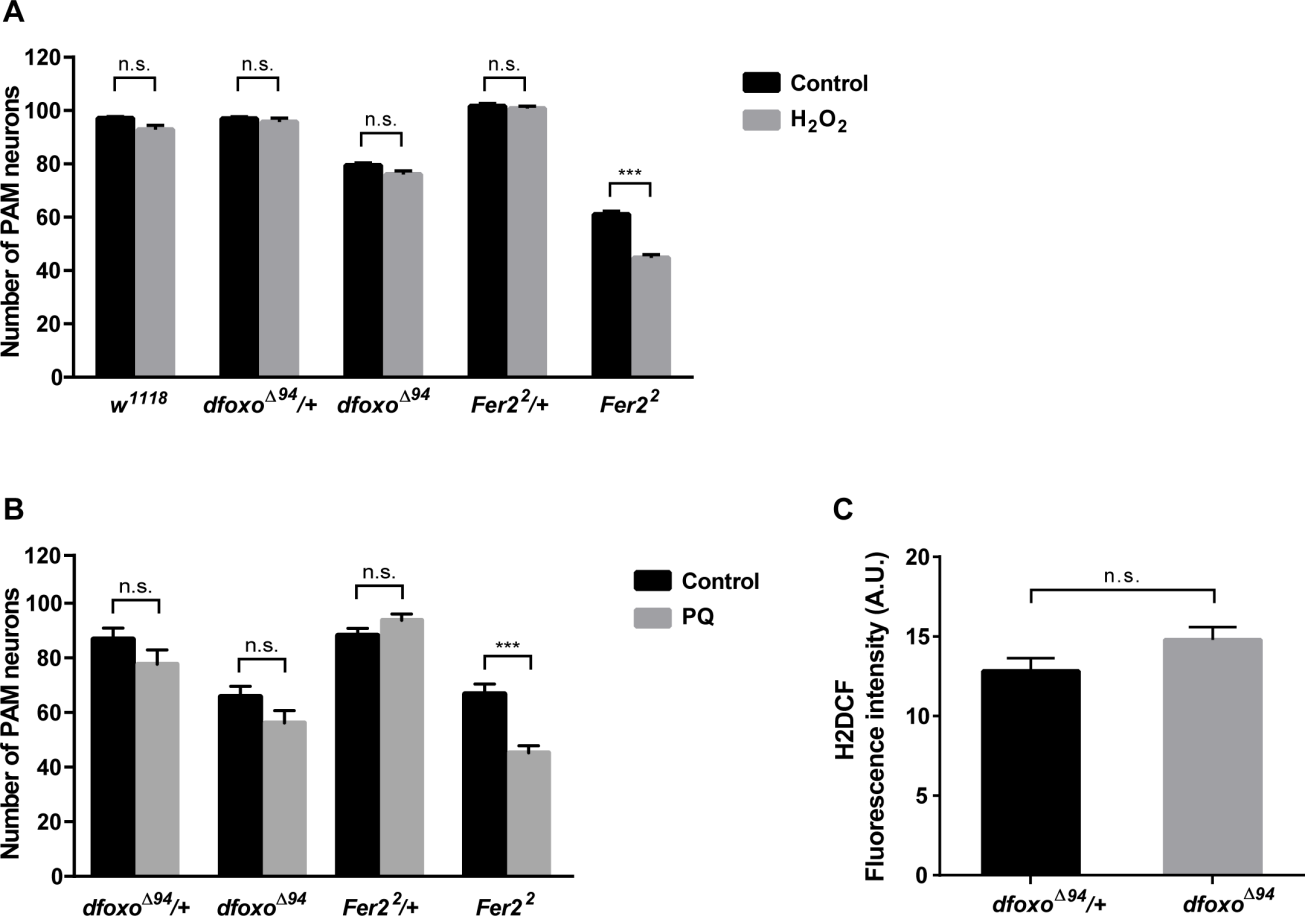
dFOXO is not required for protection of PAM neurons against acute oxidative stress. (A and B) Quantification of PAM neurons detected by anti-TH staining in the indicated genotypes after 24-h treatment with 5% H_2_O_2_ (A) or 0.2 mM paraquat (B). Mean counts per hemisphere ± SEM. *** p<0.001 by Student’s t-test (n = 14–20). (C) ROS levels in the whole brain assayed by H2DCF staining. Mean fluorescence intensity ± SEM. No significant differences are found between *dfoxo*^*Δ94*^*/+* and *dfoxo*^*Δ94*^ by Mann–Whitney *U*-test (n = 8).

To test the sensitivity of DA neurons to acute oxidative stress, 7-day-old wild-type (*w*^*1118*^), *Fer2*^*2*^*/+*, *Fer2*^*2*^, *dfoxo*^*Δ94*^/+ and *dfoxo*^*Δ94*^ flies were exposed to H_2_O_2_ for 24 h. The number of PAM neurons was assessed by anti-TH staining immediately after the treatment. Consistent with our previous results, the acute H_2_O_2_ treatment induced degeneration of PAM neurons in *Fer2*^*2*^ mutants [19]. However, no significant loss of PAM neurons was observed in *dfoxo*^*Δ94*^ mutants following the H_2_O_2_ treatment, as in *dfoxo*^*Δ94*^*/+*, *Fer2*^*2*^*/+* and *w*^*1118*^ (Fig 4A). Similarly, oxidative stress induced by paraquat caused a significant loss of PAM neurons in *Fer2*^*2*^ but not in *dfoxo*^*Δ94*^ flies (Fig 4B). Additionally, ROS levels in the brains of *dfoxo*^*Δ94*^ mutants were not different from those in *dfoxo*^*Δ94*^*/+* (Fig 4C). This result is in stark contrast with *Fer2*^*2*^ mutants, which have elevated ROS production in the brain [19]. Therefore, unlike *Fer2*, *dfoxo* is not required for the protection of PAM neurons from acute oxidative stress or for the prevention of ROS overproduction.

Metabolic stressors, such as starvation and amino acid deprivation, activate FOXO factors through a combination of post-translational modifications. Activation of dFOXO promotes genetic programs leading to energy conservation and enhanced catabolism [45]. Therefore, we next asked whether dietary restriction (DR) of amino acids could affect PAM neuron viability in *dfoxo*^*Δ94*^ or *Fer2*^*2*^ mutants. We cultured newly eclosed adult flies in the standard medium or the medium containing only sucrose and agar, and monitored PAM neurons at 7 and 21 days of age. Dietary restriction had no effect on the number of PAM neurons in both *dfoxo*^*Δ94*^ and *Fer2*^*2*^ mutants or in control flies (S7A and S7B Fig).

Collectively, these results demonstrate that *Fer2* and *dfoxo* act in parallel to protect PAM neurons from different stress conditions. *Fer2* is required for counteracting acute oxidative stress and overproduction of ROS. *dfoxo* is required for preventing the decline of PAM neuron viability during aging, yet it remains an open question what signals activate dFOXO in PAM neurons in adult flies.

### Autophagy and mitochondrial morphology are commonly impaired by *dfoxo* and *Fer2* loss of function in PAM neurons

Having analyzed differential pathways of *dfoxo* and *Fer2* activation, we next wanted to explore their downstream mechanisms involved in the control of PAM neuron viability. FOXO factors regulate autophagy in many different cell types both in vertebrates and invertebrates [23, 46]. Autophagy is a conserved process that promotes the degradation of damaged organelles and protein aggregates. Decreased autophagy is associated with premature aging and age-related neurodegenerative diseases (Reviewed in [47, 48]). The expression of several *autophagy-related genes* (*Atgs*) and, concomitantly, basal levels of autophagy have been reported to decrease with age in fly brains [49, 50]. The Atg8 protein is an essential component of autophagosome formation and remains associated with autophagosomes in all stages of autophagy [51]. RNAseq analysis of isolated PAM neurons revealed the expression of several *Atg* genes, including *Atg8*, within PAM neurons (S2 Fig). Therefore, to examine whether autophagy is dysregulated in PAM neurons in *dfoxo*^*Δ94*^ or *Fer2*^*2*^ mutants, we monitored the Atg8 protein by immunostaining with anti-Atg8 antibodies [52].

**Fig 5:**
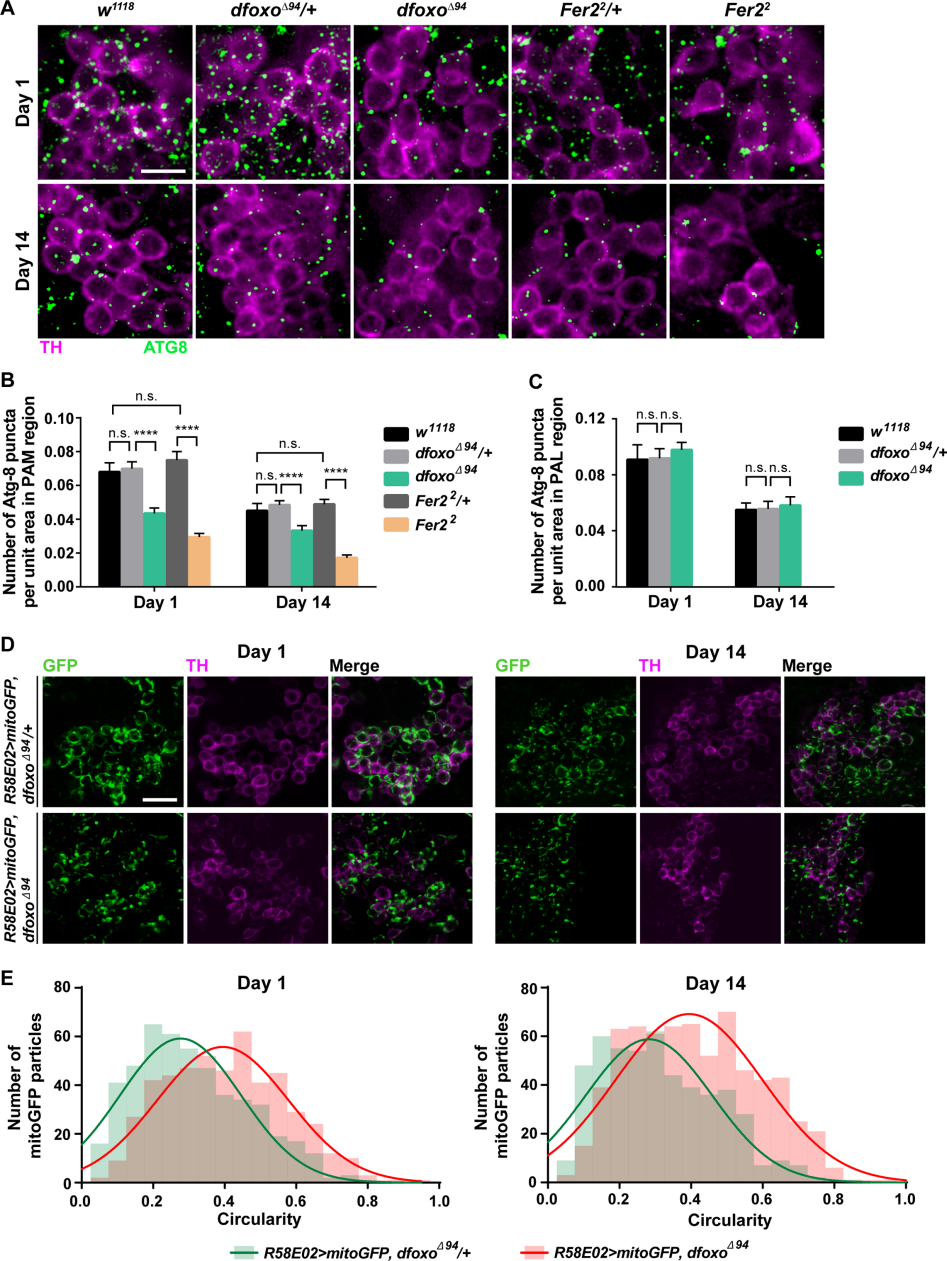
Impaired autophagy and mitophagy are the common features of *dfoxo*^*Δ94*^ *a*nd *Fer2*^*2*^ mutants. (A) Representative confocal images of Atg8 immunoreactivity in PAM neurons. Scale bar, 5 μm. Green, anti-Atg8 staining. Magenta, anti-TH staining. (B and C) Quantification of Atg8-positive autophagosomes in DA neurons in the PAM (A and B) and PAL (C) clusters in the indicated genotypes and ages. Significantly fewer autophagosomes are detected in PAM neurons in *dfoxo*^*Δ94*^ and *Fer2*^*2*^ compared to the heterozygotes. Mean ± SEM. ****p<0.0001 by ANOVA. (n = 16–20). (D) Representative images of mitochondria in PAM neurons visualized by the expression of mitoGFP in *dfoxo*^*Δ94*^/+ and *dfoxo*^*Δ94*^ flies. Green, mitoGFP. Magenta, anti-TH staining. Scale bar, 10 μm. (D) Histograms of the circularity values of mitoGFP signals. Density curves are overlaid on the histograms. The histograms of *dfoxo*^*Δ94*^ are shifted toward circularity (value of 1.0) compared to those of *dfoxo*^*Δ94*^*/+*, indicating the accumulation of more rounded mitochondria in *dfoxo*^*Δ94*^. Significant differences are found between *dfoxo*^*Δ94*^*/+* and *dfoxo*^*Δ94*^ at both day 1 and day 14 by Mann-Whitney test (p<0.0001).

Consistent with previous reports, the number of Atg8-puncta in *w*^*1118*^ showed an age-dependent reduction. In *dfoxo*^*Δ94*^ and *Fer2*^*2*^ mutants, Atg8-puncta in PAM neurons were significantly reduced in number compared to that in *w*^*1118*^ and heterozygous mutants both at 1 day and 14 days old (Fig 5A and 5B). These results indicate that *dfoxo* and *Fer2* control autophagy levels in PAM neurons. Importantly, the *dfoxo*^*Δ94*^ mutation did not affect the number of Atg8-puncta in PAL neurons (Figs 5C and S8), suggesting that autophagy control by *dfoxo* is specific to certain cell types, including PAM neurons. As *Fer2* expression is restricted to PAM and PAL neurons and a small number of other brain cells [19], *Fer2* is also likely to regulate autophagy in specific cell types including PAM neurons. However, the incomplete development of PAL neurons in *Fer2*^*2*^ mutants [19] precluded the analysis of Atg8 in this cell type.

In healthy cells, mitochondrial integrity is maintained by fission-fusion dynamics and clearance of dysfunctional mitochondria by autophagy, termed mitophagy. Several lines of evidence suggest that mitochondrial dysfunction plays a key role in pathogenesis of PD [13, 53, 54]. Previous studies have shown that FOXO factors activate mitophagy, thereby protecting DA neurons in both mammalian and fly models of PD [28, 29, 55]. Because abnormal morphology of mitochondria is intimately linked to their functional impairment, we next visualized mitochondria in PAM neurons by expressing mitochondria-targeted GFP (mitoGFP) in *dfoxo*^*Δ94*^ and *dfoxo*^*Δ94*^*/+* flies. Both 1- and 14-day-old *dfoxo*^*Δ94*^ mutants appeared to accumulate enlarged, round mitochondria in PAM neurons (Fig 5D). The analysis of mitochondrial circularity (inverse of elongation; a circularity value of 1.0 indicates a perfect circle) confirmed that mitochondria in PAM neurons in *dfoxo*^*Δ94*^ mutants were quantitatively less tubular than those in *dfoxo*^*Δ94*^*/+* at both 1 day and 14 days old (Fig 5E). Accumulation of enlarged mitochondria in PAM neurons is also a pathogenic characteristic of *Fer2*^*2*^ mutants [19]. Taken together, these results suggest that *dfoxo* and *Fer2* act in parallel pathways that converge onto the control of autophagy and mitochondrial biology in PAM neurons.

## Discussion

Here, we demonstrate that dFOXO is tissue-autonomously required for the maintenance of DA neurons in the PAM cluster during aging. We further present evidence that dFOXO and FER2 act in parallel pathways to protect PAM neurons from different forms of cellular stress. However, dFOXO and FER2 partly share downstream pathways leading to the control of autophagy and mitochondrial morphology. Thus, overexpression of one can rescue the loss of function of the other. These results highlight the notion that multiple genetic and environmental risk factors interact and affect DA neuron survival. Importantly, GWAS and functional studies in mammals implicated FOXO family TFs, including *FOXO1*, *FOXO3*, *FOXA1* and *FOXA2*, in the maintenance of DA neurons and in PD [24, 25, 27, 28]. Our results are in accordance with these studies and further suggest that *dfoxo* loss of function offers a valuable tool to study the pathogenesis of sporadic PD.

### The FOXO paradox

In mammals, although constitutive activation of FOXO3 induces loss of DA neurons in the SN, the expression of a dominant negative FOXO3 causes oxidative damage that leads to DA neuron loss. Nevertheless, both the dominant-negative form and mild activation of FOXO3 are neuroprotective in mice overexpressing α-Synuclein. Thus, FOXO3 can be protective or detrimental to DA neurons in the SN depending on its activity levels and genetic background [28]. Likewise, in *Drosophila*, previous studies have shown paradoxical roles for dFOXO in the survival of DA neurons in various PD models. Koh et al. showed that dFOXO overexpression ameliorates mitochondrial abnormality and protects DA neurons in *Pink1* null mutants [29]. Conversely, dFOXO mediates the death of DA neurons by inducing apoptosis in *DJ-1β* loss-of-function mutants and in flies overexpressing dLRRK [30, 31].

The apparent paradox concerning the role of FOXOs suggests that the activity of FOXO factors should be tightly regulated in order to exert neuroprotective function, i.e., activity levels of FOXO factors that are too high or too low are both detrimental to DA neurons. Alternatively, the differences in the reagents and experimental conditions used to examine the role of FOXOs in prior studies may have contributed to the differences in the interpretation of the results. A number of experiments in the *Drosophila* studies mentioned above used global overexpression of dFOXO and tissues other than DA neurons, such as eyes, muscles and wings, were mainly analyzed to evaluate its effect [29–31]. Furthermore, for *dfoxo* loss of function experiments, these studies used *dfoxo*^*21*^ or *dfoxo*^*25*^, which contain nucleotide transversions resulting in premature stop codons but nevertheless are not null alleles [32].

In the present study, we use a genuine null allele of *dfoxo*, *dfoxo*^*Δ94*^, to examine whether endogenous dFOXO is protective or detrimental to DA neurons [32]. Our results demonstrating that dFOXO is protective to DA neurons in the PAM cluster under basal conditions are in accordance with the report by Koh et al. Curiously, however, they observed the loss of DA neurons in the DL1 (dorso lateral 1) cluster, which corresponds to the PPL1 cluster in the nomenclature for adult DA neurons [29, 56]. Additionally, by PAM neuron-targeted constitutive and adult-restricted *dfoxo* RNAi, we show here that dFOXO expression within adult PAM neurons is required for the maintenance of PAM neurons in aged flies (Figs 2 and S4).

### Crosstalk of dFOXO and FER2 pathways

Overexpression of *dfoxo* in PAM neurons prevents the developmental impairment and age-dependent loss of PAM neurons in *Fer2*^*2*^ mutants (Fig 3D). Conversely, *Fer2* overexpression ameliorates the effect of *dfoxo*^*Δ94*^ mutation on the development and maintenance of PAM neurons (Fig 3B and 3C). Since *Fer2* and *dfoxo* do not transcriptionally regulate each other (S6C and S6D Fig), the reciprocal rescue suggests that their downstream mechanisms partly overlap. In line with this interpretation, we show that autophagy and mitochondrial morphology are commonly impaired in PAM neurons of *dfoxo*^*Δ94*^ and *Fer2*^*2*^ mutants (Fig 5).

Mounting evidence indicates that FOXO factors regulate autophagy by controlling the expression of *Atg* genes in flies and mammals [23, 57]. FOXOs also regulate factors controlling mitophagy and mitochondrial remodeling in mammals [55, 58]. Therefore, dFOXO may regulate autophagy and mitophagy in PAM neurons, although dysregulation in autophagy and mitochondrial morphology in *dfoxo*^*Δ94*^ could be secondary effects of cellular damage. Uncovering genetic pathways downstream of dFOXO and FER2 and how they intersect will yield valuable information, especially because our results suggest that targeted overexpression of *dfoxo* or *Fer2* in DA neurons may confer protection against DA neuron demise in various genetic models of PD.

Consistent with the known role of PAM neurons in controlling locomotion [10, 19], startle-induced climbing ability in *dfoxo*^*Δ94*^ and *Fer2*^*2*^ mutants is significantly improved by the expression of *dfoxo* with *R58E02-GAL4* (S6A Fig). However, *R58E02>dfoxo* does not rescue the shortened lifespan of *dfoxo*^*Δ94*^ and even further reduces the lifespan of *Fer2*^*2*^ (S6B Fig). Therefore, neuroprotective role of dFOXO is independent of its role in longevity regulation. Many fly models of PD show lifespan shortening [14, 18, 35–37], which is likely caused by the systemic effect of mitochondrial impairment and/or elevated oxidative stress levels rather than DA neuron demise. Lifespan shortening of *dfoxo*^*Δ94*^ and *Fer2*^*2*^ mutants may be similarly attributed to the impairment in mitochondrial biology or (in the case of *Fer2*^*2*^) oxidative stress regulation in cells other than PAM neurons.

Given that mitochondrial dysfunction and oxidative stress are tightly linked and both implicated in neurodegeneration, it is surprising that we found no evidence that PAM degeneration in *dfoxo*^*Δ94*^ is associated with chronic or acute oxidative stress, unlike *Fer2*^*2*^ mutants (Fig 4). Our results also show no evidence that amino acid intake during adulthood is relevant for survival of PAM neurons (S7 Fig). Then, how is dFOXO signaling activated during adulthood to promote PAM neuron survival in aged flies? Aging is associated with loss of proteostasis [59, 60] and FOXOs play a key role in cellular proteostasis. Consistent with the findings in other tissues [49, 50, 61], autophagy levels in PAM neurons decrease with age, and this is accelerated in *dfoxo*^*Δ94*^ (Fig 5A and 5B). Thus, age-dependent decrease in basal activity of autophagy might be an intracellular stress signal that leads to the activation of dFOXO in PAM neurons.

Our study reveals an unexpected crosstalk between two pathways mediated by two TFs, dFOXO and FER2, in the development and maintenance of DA neurons in the PAM cluster. Importantly, both genes are also required for the proper development of PAM neurons. This is in line with the fact that several mammalian TFs required for DA neuron development play critical roles in the maintenance of adult midbrain DA neurons [62–66]. dFOXO homologs FOXA1 and A2 fall within this category [25–27], suggesting that TFs having dual roles in the development and maintenance of DA neurons is an evolutionarily conserved mechanism of neuroprotection. Furthermore, our data suggest that loss of *dfoxo* expression before adulthood has lasting detrimental effect on the survival of PAM neurons in aging flies, which may be partly regulated non-cell-autonomously by dFOXO in the larval fat body or in other tissues. In conclusion, our study provides a starting point to investigate TF networks underlying the link between aberrant neural development and neurodegeneration, which will present new opportunities to better understand the etiology of sporadic PD.

## Materials and methods

### *Drosophila* stocks and maintenance

The following lines were obtained from the Bloomington Stock Center (BDSC) (Indiana University, Bloomington, IN): *UAS-RedStinger* (# 8546), *UAS-dfoxo RNAi-2* (# 32427), and *UAS-dfoxo RNA -3 (# 32993)*. *UAS-dfoxo RNAi-1* (v30557) was obtained from the Vienna Drosophila Resource Center. *UAS-dfoxo RNAi* lines were characterized in previous studies [39, 40], and for simplicity referred to as RNAi-1 to -3 in this paper. *Mi{ET1}Fer2*^*MB09480*^ (referred to as *Fer2*^*2*^) and *UAS-mitoGFP*; *UAS-miR Fer2-N*, *UAS-miR Fer2-5*, *UAS-Fer2Flag*, *tubulin-GAL80*^*ts*^, *Fer2-Gal4* and *R58E02-GAL4* lines were previously described [19, 34]. *dfoxo*^*Δ94*^ was a kind gift from L. Partridge [32] and *UAS-dfoxo* was from E. Hafen [42]. *0*.*68Lsp2-GAL4* was a gift from B. Dauwalder [67]. All flies were maintained on standard cornmeal-agar food at 25°C in a 12 h:12 h light-dark cycle and controlled humidity.

### Immunohistochemistry

Immunostaining with an anti-TH antibody with or without other antibodies was performed on whole fly brains as previously described [19] with minor modifications. Briefly, flies were decapitated, and the heads were fixed in 4% paraformaldehyde + 0.3% Triton X-100 for 1 h on ice and washed three times with PBST-0.3 (PBS, 0.3% Triton X-100). The cuticle was partially removed, and the partly opened heads were blocked in blocking solution (5% normal goat serum, PBS, 0.3% Triton X-100) for 1 h at room temperature on a rocking platform. Subsequently, the samples were incubated with the primary antibodies for two overnights at 4°C. After 3 washes, the samples were then incubated with Alexa Fluor-conjugated secondary antibodies overnight at 4°C. Then, the remaining cuticle and trachea were removed and the brains were mounted in Vectashield (Vector Laboratories). The primary antibodies and the concentrations applied in this study were as follows: rabbit polyclonal anti-tyrosine hydroxylase (ab152, Millipore) 1:100, mouse monoclonal antibody nc82 (Developmental Studies Hybridoma Bank) 1:100, rabbit anti-Atg8 (kindly provided by K. Köhler) [52] 1:200 and polyclonal rabbit anti-GFP (A6455, Invitrogen) 1:250.

### Microscopy and image analysis

Fly brains were scanned using a Leica TCS SP5 confocal microscope. Quantification of DA neurons, Atg8-positive autophagosomes and mitochondrial circularity was performed using ImageJ software (NIH).

To obtain the number of DA neurons, anti-TH-positive neurons or the cells expressing RedStinger driven by *R58E02-GAL4*, cells were counted manually through Z-stacks of confocal images using the cell counter plugin of ImageJ. PAM neuron counts were scored blindly. To count the number of Atg8-positive autophagosomes in DA neurons in the PAM and PAL clusters, Atg8-puncta colocalized with TH-positive PAM neurons were counted automatically using the particle analysis plugin in ImageJ. To measure the circularity of mitochondria in PAM clusters, mitoGFP-positive objects within the TH-positive PAM neurons were analyzed using the particle analysis plugin with the circularity analysis tool in ImageJ.

### Startle-induced climbing assay

For each experimental group, 20 male flies were briefly anesthetized with CO_2_ and put into a 100-ml graduated glass cylinder, which was divided into 5 equal parts and marked from 0 to 5 from the bottom to the top. Flies were left to recover from the CO_2_ exposure for 1 h before the assay. The climbing assay was performed at ZT2 (2 h after lights-on in 12 h:12 h light-dark cycles) to avoid circadian variations in the locomotor activities of the flies, following the procedure previously described [19]. Briefly, the cylinder was tapped on a flexible pad to collect all of the flies at the bottom, and then the flies were allowed to climb up the wall of the cylinder for 20 s. The assay was video recorded and analyzed using the VLC media player. The number of flies that climbed up to each zone within 20 s was counted manually and used to calculate the climbing index (CI) using the following formula: CI = (0 x n_0_ + 1 x n_1_ + 2 x n_2_ + 3 x n_3_ + 4 x n_4_ + 5 x n_5_) / n_total_, where n_total_ is the total number of flies and n_x_ is the number of flies that reached zone X. Each experiment consisted of 3 trials, and 3 independent experiments were performed for each condition. The mean values of 3 independent experiments are shown.

### Lifespan analysis

Flies were collected immediately after hatching and kept at 25°C in 12 h:12 h light-dark cycles and controlled humidity on standard fly food. For each experimental group, male flies were placed into vials containing food at a density of 20 flies per vial, and in total, 5 vials (n = 100 flies) were maintained for each group. Flies were transferred onto fresh food 3 times a week, and the number of live flies was recorded at every transfer.

### Treatment with oxidative reagents

5-day-old flies were placed into empty vials and starved for 6 h. The flies were then transferred into vials containing Drosophila instant food (Formula 4–24 Instant Drosophila Medium, Carolina Biological Supply Company, USA) containing 5% H_2_O_2_, 0.2 mM paraquat or vials containing Drosophila instant food only for control and kept for 24 h. All vials were kept in a humid box at 25°C during the treatment. Immediately after the treatment, the flies were decapitated, and anti-TH-staining was performed as described above.

### ROS detection

To measure the level of ROS produced in the fly brain, a cell-permeable probe, 2’7’-dichlorofluorescin diacetate (H2DCF), was used. H2DCF is de-esterified intracellularly and turns into a highly fluorescent form, 2’7’-dichlorofluorescin, upon oxidation, allowing for the detection of ROS. The detailed experimental protocol was described in Owusu-Ansah et al. [68]. The fluorescence intensity in the entire central brain was measured using FIJI software [69] by performing a Z-SUM projection across the corresponding Z-stacks, as previously described [19].

### RT-qPCR

Total RNA was isolated from whole fly heads using TRIzol reagent (Life Technologies). Then, cDNA was synthesized by reverse transcription of the mRNAs using oligo(dT) primers and was then used as templates for qPCR, which was performed as previously described [70]. mRNA levels of *Fer2* were normalized to those of the housekeeping gene, *elongation factor 1b* (*Ef1b*).

### Dietary restriction

Newly eclosed male flies were used for this experiment. All flies were kept at 25°C, in 12 h:12 h light-dark cycles and controlled humidity. For dietary restriction (DR), flies were transferred into the vials containing the medium composed of 5% sucrose and 2% agar. For standard diet (SD), flies were kept in the vials containing *Drosophila* instant food (Formula 4–24 Instant Drosophila Medium, Carolina Biological Supply Company, USA). One set of flies was kept for 7 days, and second set of flies were kept for 21 days in DR or SD conditions. n = 6–8 per genotype per condition.

### Statistics

Statistical analyses were performed using GraphPad Prism 6 software. Lifespan data were subjected to survival analysis (log-rank tests) using the Mantel-Haenszel approach (also called the Mantel-Cox method). For all the other data, to compare two experimental groups of normally distributed data sets, a two-tailed Student’s *t*-test was used. To compare two groups of non-normally distributed data sets, non-parametric statistics (Mann–Whitney U-test) were used. ANOVA with a Tukey’s HSD post-hoc test was used to compare two groups with more than one factor to be considered and to compare three or more groups. For all of the experiments, the level of significance was set at p<0.05.

## Supporting information

S1 Fig Loss of PAM neurons in *dfoxo*^*Δ94*^ mutants. (A) Representative images of the PAM neurons visualized by driving expression of RedStinger (magenta) with *R58E02-GAL4* in *dfoxo*^*Δ94*^ and *dfoxo*^*Δ94*^*/+* at the indicated ages. Scale bar, 20 μm. (B) Quantification of (A) indicates a significant age-dependent loss of PAM neurons. Mean ± SEM. *p<0.05 and **** p<0.0001 by ANOVA (n = 20).
(TIF)

S2 Fig Transcripts expression in PAM neurons. mRNA expression of isolated PAM neurons was analyzed by RNA-seq. Expression levels of some of the relevant transcripts are shown as mean RPKM (Reads Per Kilobase of transcript per Million mapped reads) of triplicate samples. Error bars represent SEM. DA neuron markers, *ple* and *Ddc*, as well as *dfoxo*, *Fer2*, and several *Atg* genes are expressed in PAM neurons. mRNA of glia-specific *gcm* was not detected.
(TIF)

S3 Fig *R58E02-GAL4* is expressed in the fat body of young adult flies. (A) The abdominal fat body of adult flies expressing *UAS-GFP-NLS* with *R58E02-GAL4* or *Fer2-GAL4* was dissected at the indicated ages and double stained with anti-GFP antibodies and Nile red. *R58E02/+* and *Fer2-GAL4/+* were driver-only negative controls (4 days old). Scale bar, 100 μm. (B) The fat body of 3rd instar larvae was stained with Nile red. Larval fat body-specific *0*.*68Lsp2-GAL4* was used to drive *UAS-GFP-NLS* reporter as a positive control. No expression of *R58E02-GAL4* or *Fer2-GAL4* is detected in larval fat body. Scale bar, 250μm.
(TIF)

S4 Fig dFOXO knockdown with *R58E02-GAL4* leads to the loss of PAM neurons. Mean number of PAM neurons following *dfoxo* knockdown with *UAS-dfoxo RNAi-1*, *-2*, or *-3* driven by *R58E02-GAL4* (n = 14–20). Error bar, SEM. *p<0.05, ***p<0.001 by ANOVA.
(TIF)

S5 Fig Viability of DA neurons in the PAL cluster is unaffected by *dfoxo*-*Fer2* double loss-of-function. PAL neurons were detected by anti-TH staining in the indicated genotypes and age. Mean counts of PAL neurons per hemisphere, error bars represent SEM. No significant differences are found by ANOVA.
(TIF)

S6 Fig *dfoxo* and *Fer2* act in parallel to protect PAM neurons. (A) Startle-induced climbing response of 14-day-old flies. *UAS-Fer2* and *UAS-dfoxo* expression driven by *R58E02-GAL4* significantly improved the climbing ability of *dfoxo*^*Δ94*^ and *Fer2*^*2*^ flies, respectively. Mean climbing index ± SEM. **p<0.01, ***p<0.001 and ****p<0.0001 by Mann-Whitney U-test. (B) Lifespan assay. *Fer2* expression driven by *R58E02-GAL4* significantly improves lifespan of *dfoxo*^*Δ94*^ mutants (p<0.0001, log-rank test), whereas *dfoxo* expression reduces the lifespan of *Fer2*^*2*^ (p<0.0001, log-rank test). *R58E02 > dfoxo* does not increase the lifespan of *dfoxo*^*Δ94*^ mutants (p>0.05, log-rank test). (C) *Fer2* mRNA levels in the heads of 7-day-old flies quantified by qPCR. No differences in the *Fer2* mRNA levels are found between *w*^*1118*^, *dfoxo*^*Δ94*^*/+* and *dfoxo*^*Δ94*^. (D) Integrative Genomics Viewer (IGV) screenshots of *Fer2*::*V5* ChIP-seq analysis on the *dfoxo* promoter and the *CG31221* gene. *CG31221* is shown as a representative FER2 binding peak (arrow). ChIP-seq was performed on *Fer2*^*1*^ mutant flies rescued by expressing a V5-tagged *Fer2* genomic transgene (*Fer2*::*V5*) and on *w*^*1118*^ flies (negative control), which identified approximately 200 FER2 binding peaks in the fly brain but not in the *dfoxo* gene.
(TIF)

S7 Fig Amino acid deprivation does not affect PAM neuron survival. Mean PAM neuron counts of the flies cultured on standard food (standard diet, SD) or on the media containing only sucrose and agar (dietary restriction, DR) at 7 days old (A) and 21 days old (B). Error bars represent SEM. No significant differences are found between SD and DR by ANOVA in all gentotypes.
(TIF)

S8 Fig Atg8-positive autophagosomes in PAL neurons. Representative confocal images of Atg8 immunoreactivity in PAL neurons in 1- and 14-day-old flies of indicated genotypes. Scale bar, 10 μm. Green, anti-Atg8 staining. Magenta, anti-TH staining.
(TIF)

S1 Text Supporting materials and methods. Materials and methods for Chromatin immunoprecipitation coupled to sequencing (ChIP-seq) and RNA-seq analysis of isolated PAM neurons.
(DOCX)
