# Supplementary figures and images for: Parallel roles of transcription factors dFOXO and FER2 in the development and maintenance of dopaminergic neurons
Source: PLoS Genet. 2018 Mar 12;14(3):e1007271. doi: 10.1371/journal.pgen.1007271 (PMC5864087; doi:10.1371/journal.pgen.1007271)

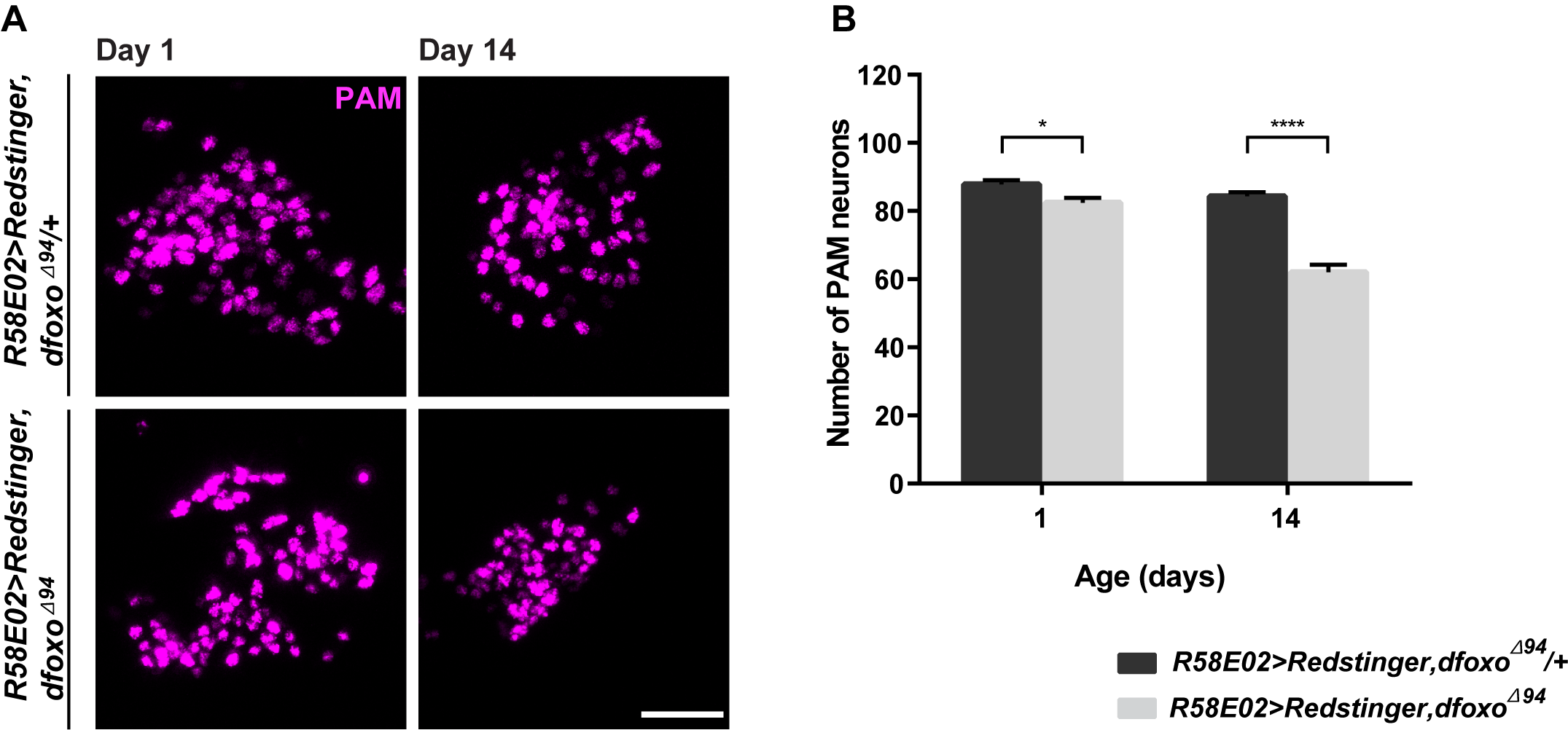

Supplement: S1 Fig — (A) Representative images of the PAM neurons visualized by driving expression of RedStinger (magenta) with R58E02-GAL4 in dfoxoΔ94 and dfoxoΔ94/+ at the indicated ages. Scale bar, 20 μm. (B) Quantification of (A) indicates a significant age-dependent loss of PAM neurons. Mean ± SEM. *p<0.05 and **** p<0.0001 by ANOVA (n = 20). (TIF) [file pgen.1007271.s001.tif]

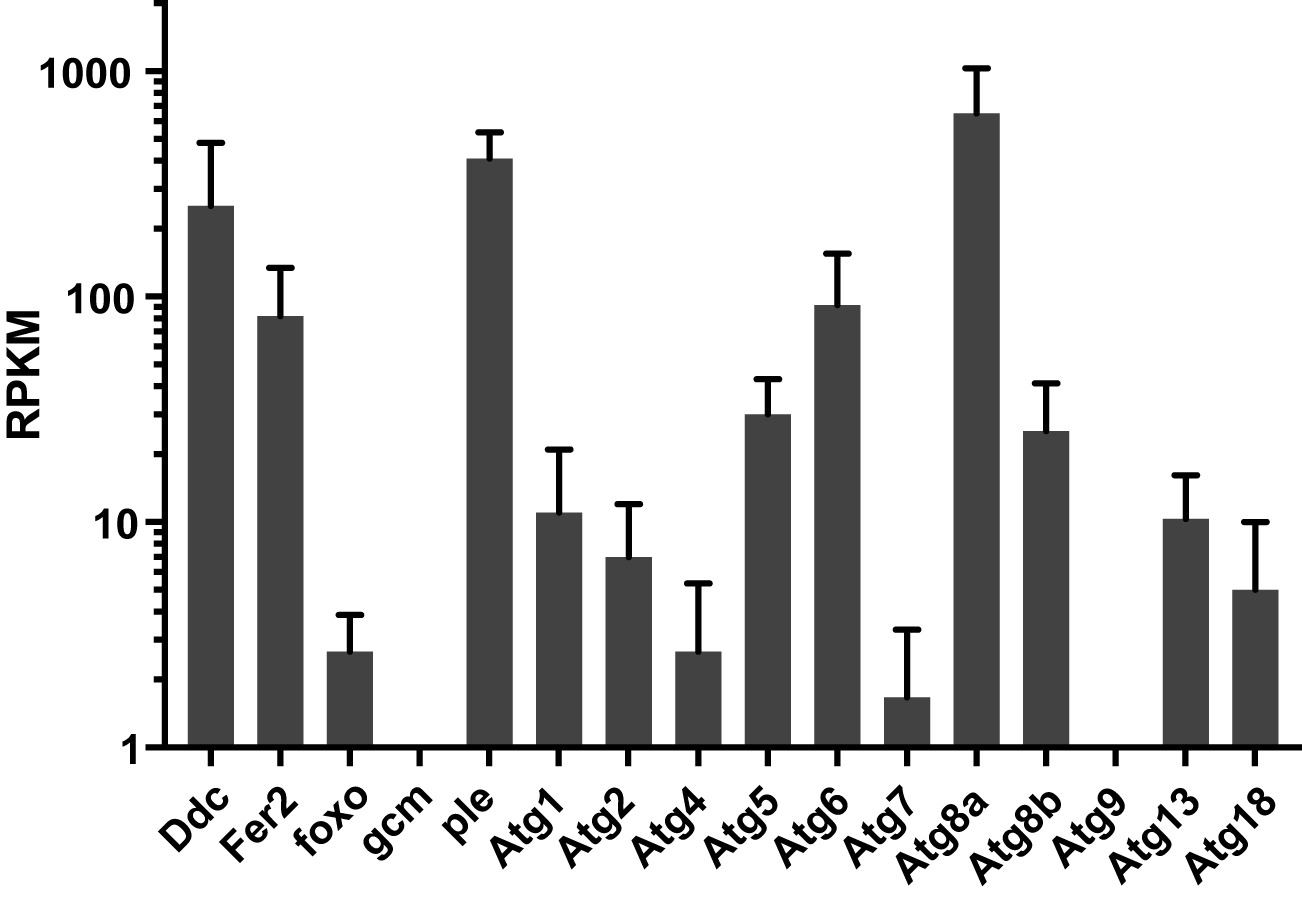

Supplement: S2 Fig — mRNA expression of isolated PAM neurons was analyzed by RNA-seq. Expression levels of some of the relevant transcripts are shown as mean RPKM (Reads Per Kilobase of transcript per Million mapped reads) of triplicate samples. Error bars represent SEM. DA neuron markers, ple and Ddc, as well as dfoxo, Fer2, and several Atg genes are expressed in PAM neurons. mRNA of glia-specific gcm was not detected. (TIF) [file pgen.1007271.s002.tif]

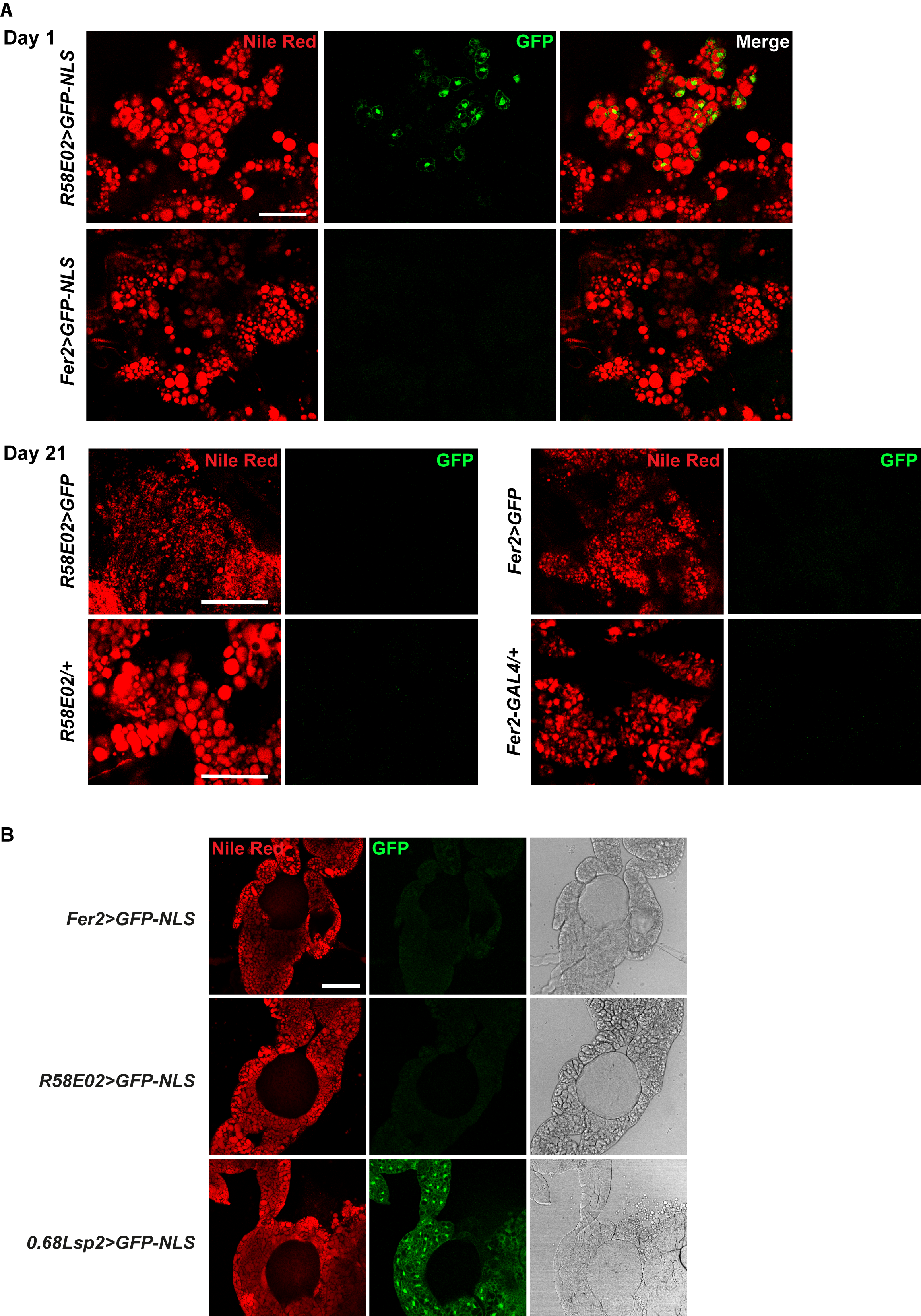

Supplement: S3 Fig — (A) The abdominal fat body of adult flies expressing UAS-GFP-NLS with R58E02-GAL4 or Fer2-GAL4 was dissected at the indicated ages and double stained with anti-GFP antibodies and Nile red. R58E02/+ and Fer2-GAL4/+ were driver-only negative controls (4 days old). Scale bar, 100 μm. (B) The fat body of 3rd instar larvae was stained with Nile red. Larval fat body-specific 0.68Lsp2-GAL4 was used to drive UAS-GFP-NLS reporter as a positive control. No expression of R58E02-GAL4 or Fer2-GAL4 is detected in larval fat body. Scale bar, 250μm. (TIF) [file pgen.1007271.s003.tif]

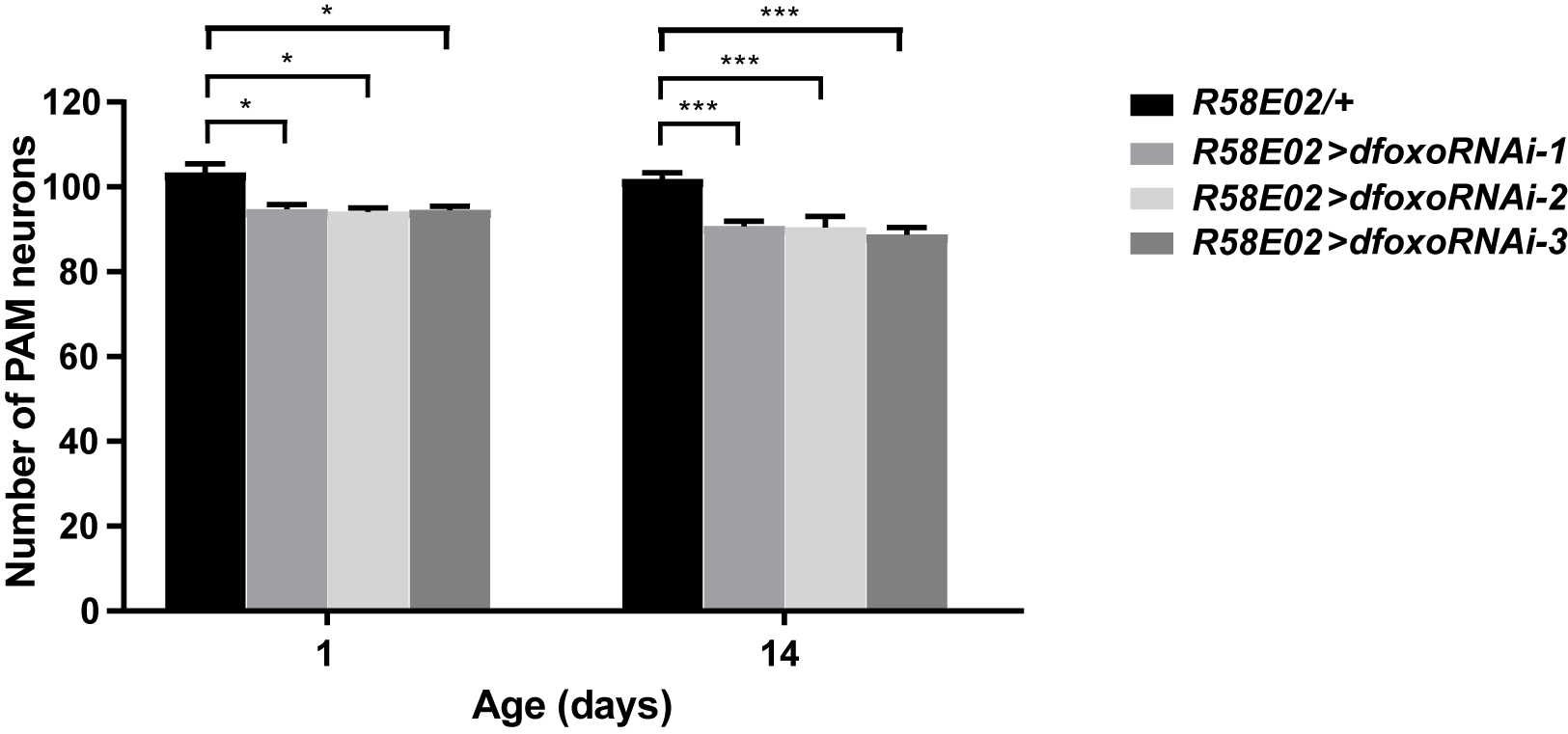

Supplement: S4 Fig — Mean number of PAM neurons following dfoxo knockdown with UAS-dfoxo RNAi-1, -2, or -3 driven by R58E02-GAL4 (n = 14–20). Error bar, SEM. *p<0.05, ***p<0.001 by ANOVA. (TIF) [file pgen.1007271.s004.tif]

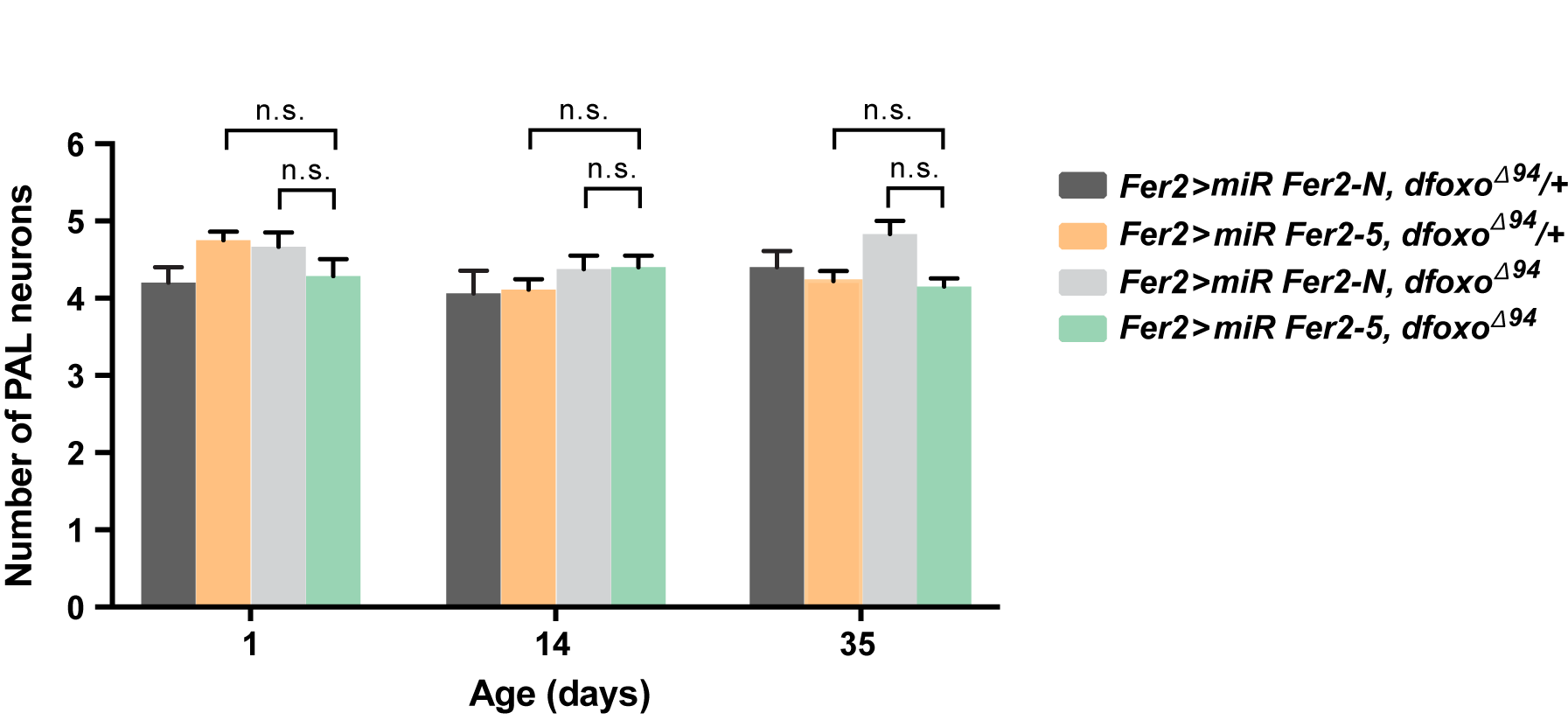

Supplement: S5 Fig — PAL neurons were detected by anti-TH staining in the indicated genotypes and age. Mean counts of PAL neurons per hemisphere, error bars represent SEM. No significant differences are found by ANOVA. (TIF) [file pgen.1007271.s005.tif]

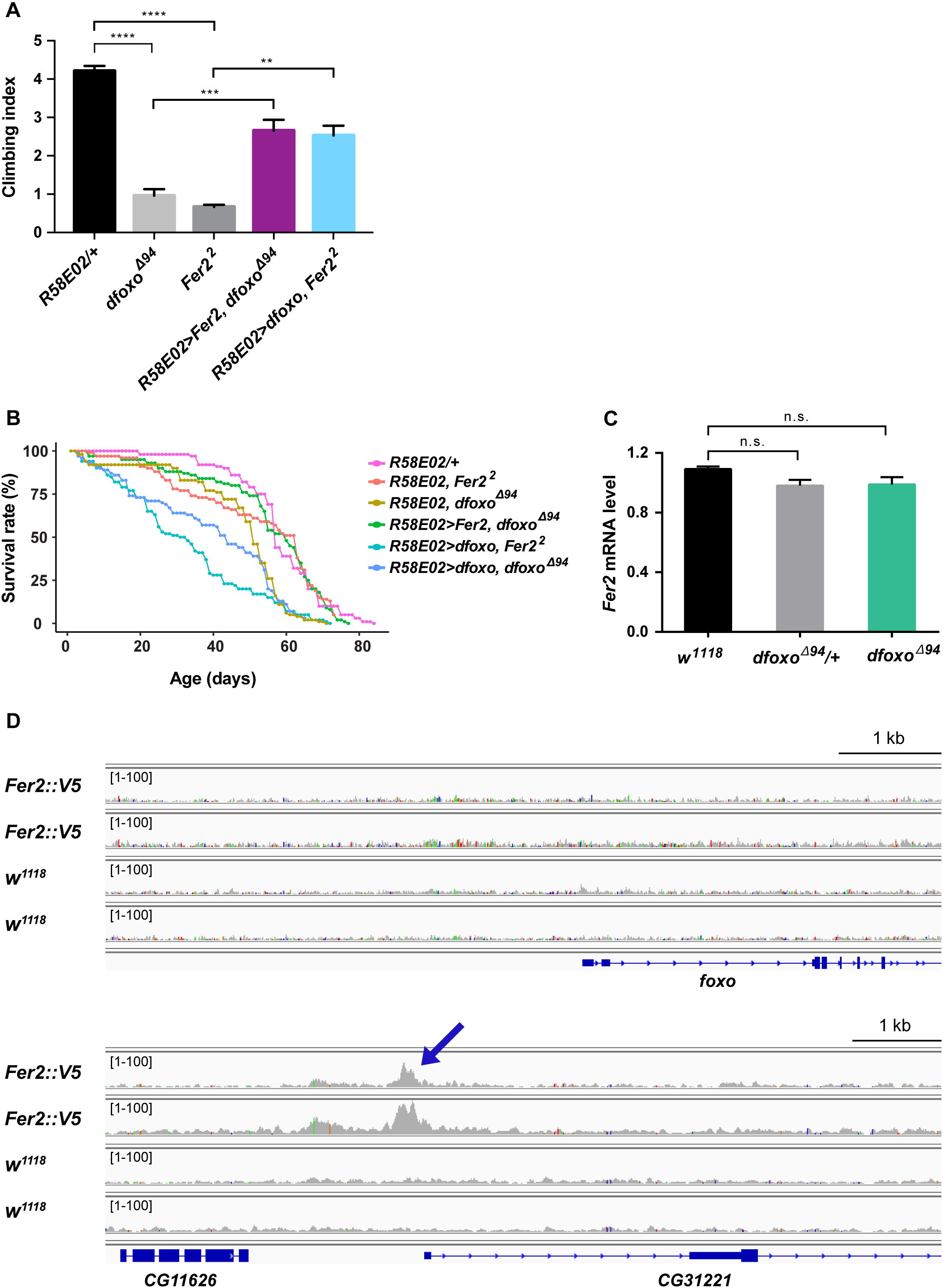

Supplement: S6 Fig — (A) Startle-induced climbing response of 14-day-old flies. UAS-Fer2 and UAS-dfoxo expression driven by R58E02-GAL4 significantly improved the climbing ability of dfoxoΔ94 and Fer22 flies, respectively. Mean climbing index ± SEM. **p<0.01, ***p<0.001 and ****p<0.0001 by Mann-Whitney U-test. (B) Lifespan assay. Fer2 expression driven by R58E02-GAL4 significantly improves lifespan of dfoxoΔ94 mutants (p<0.0001, log-rank test), whereas dfoxo expression reduces the lifespan of Fer22 (p<0.0001, log-rank test). R58E02 > dfoxo does not increase the lifespan of dfoxoΔ94 mutants (p>0.05, log-rank test). (C) Fer2 mRNA levels in the heads of 7-day-old flies quantified by qPCR. No differences in the Fer2 mRNA levels are found between w1118, dfoxoΔ94/+ and dfoxoΔ94. (D) Integrative Genomics Viewer (IGV) screenshots of Fer2::V5 ChIP-seq analysis on the dfoxo promoter and the CG31221 gene. CG31221 is shown as a representative FER2 binding peak (arrow). ChIP-seq was performed on Fer21 mutant flies rescued by expressing a V5-tagged Fer2 genomic transgene (Fer2::V5) and on w1118 flies (negative control), which identified approximately 200 FER2 binding peaks in the fly brain but not in the dfoxo gene. (TIF) [file pgen.1007271.s006.tif]

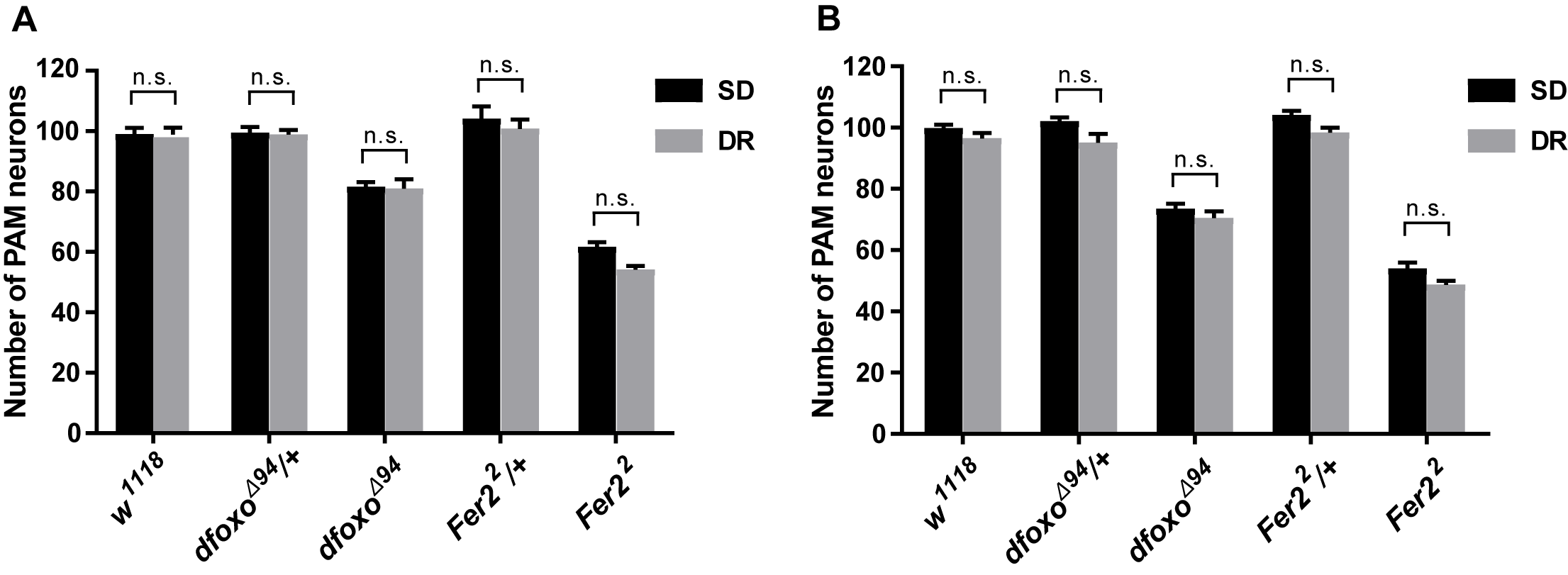

Supplement: S7 Fig — Mean PAM neuron counts of the flies cultured on standard food (standard diet, SD) or on the media containing only sucrose and agar (dietary restriction, DR) at 7 days old (A) and 21 days old (B). Error bars represent SEM. No significant differences are found between SD and DR by ANOVA in all gentotypes. (TIF) [file pgen.1007271.s007.tif]

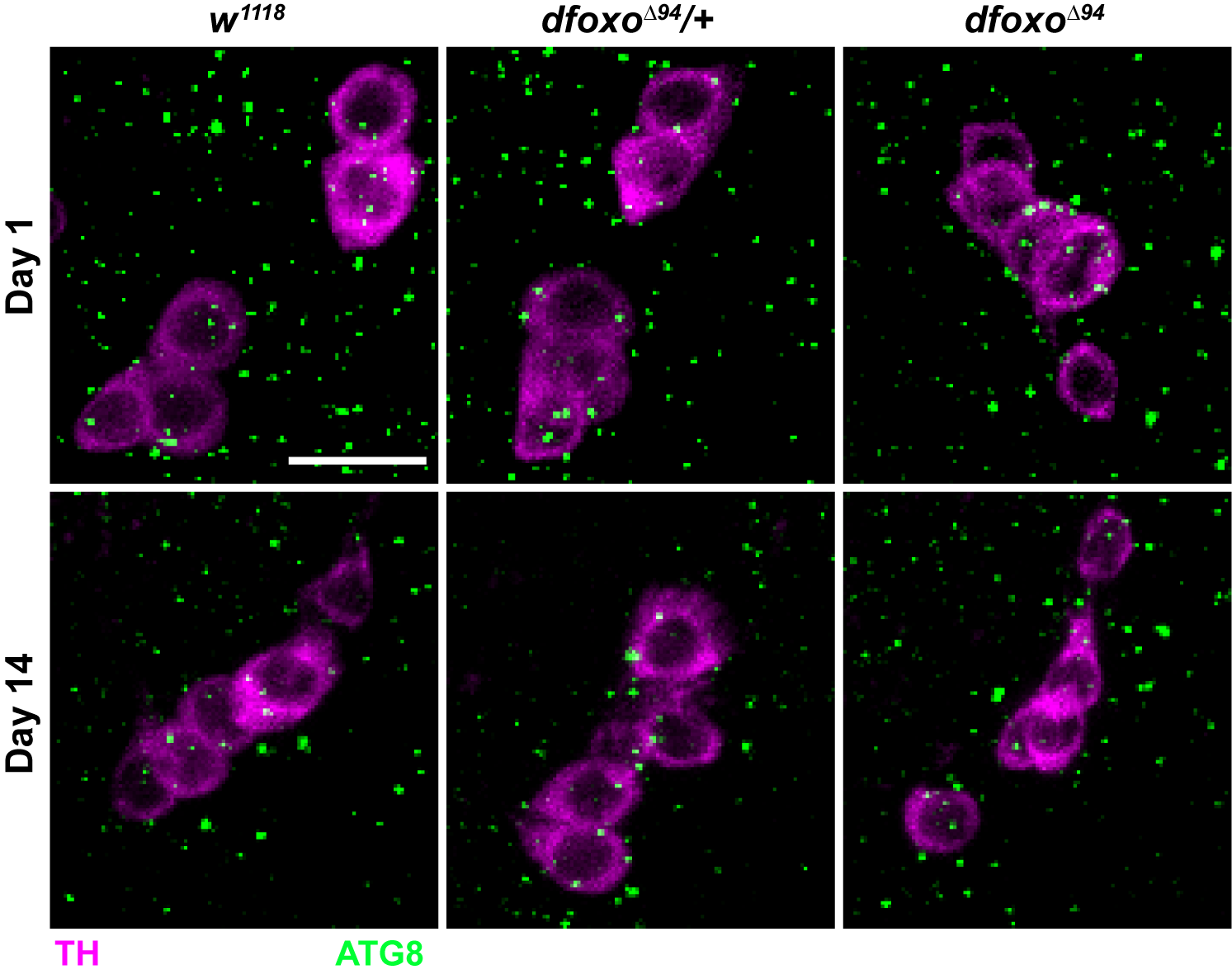

Supplement: S8 Fig — Representative confocal images of Atg8 immunoreactivity in PAL neurons in 1- and 14-day-old flies of indicated genotypes. Scale bar, 10 μm. Green, anti-Atg8 staining. Magenta, anti-TH staining. (TIF) [file pgen.1007271.s008.tif]
