# Supplementary material for: Parallel roles of transcription factors dFOXO and FER2 in the development and maintenance of dopaminergic neurons
Source: PLoS Genet. 2018 Mar 12;14(3):e1007271. doi: 10.1371/journal.pgen.1007271 (PMC5864087; doi:10.1371/journal.pgen.1007271)
Supplement: S1 Text — Materials and methods for Chromatin immunoprecipitation coupled to sequencing (ChIP-seq) and RNA-seq analysis of isolated PAM neurons. (DOCX) [file pgen.1007271.s009.docx]

**S1 Text. Supporting Materials and Methods**

**Chromatin immunoprecipitation coupled to sequencing (ChIP-seq)**

ChIP was performed based on previous protocols [71, 72] on *Fer2^1^* mutant flies expressing a V5-tagged *Fer2* genomic transgene (*Fer2::V5*) and on *w^1118^* flies (negative control). Briefly, 14-day-old flies were collected 2 h before lights-on (ZT22), frozen in liquid nitrogen and kept at -80°C until processed. Heads were collected using metal sieves, and then 1 ml of heads was ground in liquid nitrogen and homogenized 30 times in 5 ml of NE Buffer (15 mM HEPES pH 8, 10 mM KCl, 0.1 mM EDTA, 0.5 mM EGTA, 350 mM sucrose, 0.1% Tween 20, 5 mM MgCl2, 1 mM DTT, 1 mM PMSF plus Protease inhibitor cocktail (Roche Cat. No. 11 873 580 001)) with 1% formaldehyde. Fixation was performed for 10 min at RT and quenched with 125 mM glycine for 5 min at RT. Homogenate was filtered through a 100-µm nylon mesh filter (Fisher Scientific 22363549), and nuclei were collected by centrifugation at 800 x *g* for 5 min at 4°C. Nuclei were washed three times, resuspended in 1.5 ml of cold RIPA buffer (25 mM Tris-HCl pH 8, 150 mM NaCl, 0.5% sodium deoxycholate, 0.1% SDS, 1% NP-40, 0.5 mM DTT plus Protease inhibitor cocktail) and sonicated using a Bioruptor sonicator for 18 min (30 s ON/30 s OFF). Sonicated chromatin was then centrifuged at 10000 x *g* for 10 min at 4°C to remove cell debris. The supernatant was pre-cleared with agarose beads (1 h, 4°C) and incubated with 50 µl of saturated anti-V5 beads (Sigma A7345) overnight at 4°C. Beads were washed twice with low-salt buffer (20 mM Tris pH 8, 150 mM NaCl, 2 mM EDTA, 0.1% SDS, 1% Triton X-100), twice with high-salt buffer (20 mM Tris pH 8, 500 mM NaCl, 2 mM EDTA, 0.1% SDS, 1% Triton X-100), twice with LiCl buffer (10 mM Tris pH 8, 250 mM LiCl, 1 mM EDTA, 1% sodium deoxycholate, 1% NP-40) and twice with TE buffer (10 mM Tris-HCl pH 8, 1 mM EDTA). DNA was eluted in the V5 Elution Buffer (10 mM HEPES, 1.5 mM MgCl2, 0.25 mM EDTA, 20% glycerol, 250 mM KCl, 0.3% NP-40, 0.5 mg/ml V5 peptide) and incubated with RNase (30 min, 37°C) and proteinase K (overnight, 65°C). The following day, DNA was purified with a MinElute PCR purification kit (Qiagen 28004). Libraries were prepared with 0.5 to 0.7 ng of ChIP-enriched DNA as starting material and processed using the Illumina TruSeq ChIP kit according to manufacturer specifications. Libraries were validated on a Tapestation 2200 (Agilent) and a Qubit fluorometer (Invitrogen – Thermo Fisher Scientific). Pools of 8 libraries were loaded at 7 pM for clustering. Single reads of 50 bp were generated using the TruSeq SBS HS v3 chemistry on an Illumina HiSeq 2500 sequencer.

**RNA-seq analysis of isolated PAM neurons**

Cell isolation, RNA extraction, amplification of poly(A)-tailed RNAs and sequencing were performed as described previously [73]. Briefly, 10 brains of 14-day-old flies were dissected and optic lobes were removed. Dissected brains were digested with L-cystein-activated papain (50 units/ml in dissecting saline, Worthington, NJ ) for 25 min at room temperature and subsequently triturated with flame-rounded P1000 filter tip and P200 filter tip sequentially. Approximately 200 PAM neurons labeled by *R58E02-GAL4,* *UAS-RedStinger* were collected to prepare a library for sequencing on an Illumina HiSeq 2500. Biological triplicate samples were sequenced at the iGE3 Genomics Platform of the University of Geneva ([https://ige3.genomics.unige.ch](https://ige3.genomics.unige.ch/)). The mapping and quantitative analysis were performed using the online High-Throughput Sequencing portal of the EPFL (École Polytechnique Fédérale de Lausanne) Bioinformatics and Biostatistics core facility (HTSstation, <http://htsstation.epfl.ch/>).

**Nile red and antibody staining of the fat body**

Abdominal fat body was dissected as described in [74]. Heads and abdominal parts were fixed and stained with anti-GFP antibody as described [19]. Following secondary antibody incubation, lipids were stained in the first wash for 1h using 2 µg/ml Nile red (Sigma no. 19123, stock solution 1 mg/ml in DMSO). Samples were washed two more times for 30 min. Heads were dissected and abdomens were mounted whole in Vectashield mounting medium (Vector Laboratories). Images were recorded on a Leica TCS SP5 confocal microscope. The fat body of 3^rd^ instar larvae was stained with 20 µg/ml Nile Red in PBS for 30 min at room temperature.

**Supporting Information References**

71. Meireles-Filho AC, Bardet AF, Yanez-Cuna JO, Stampfel G, Stark A. cis-regulatory requirements for tissue-specific programs of the circadian clock. Curr Biol. 2014;24(1):1-10.

72. Zhou J, Yu W, Hardin PE. ChIPping away at the Drosophila clock. Methods Enzymol. 2015;551:323-47.

73. Kozlov A, Jaumouille E, Machado Almeida P, Koch R, Rodriguez J, Abruzzi KC, et al. A Screening of UNF Targets Identifies Rnb, a Novel Regulator of Drosophila Circadian Rhythms. J Neurosci. 2017;37(28):6673-85.

74. Lee JH, Bassel-Duby R, Olson EN. Heart- and muscle-derived signaling system dependent on MED13 and Wingless controls obesity in Drosophila. Proc Natl Acad Sci U S A. 2014;111(26):9491-6.
